# Method of establishing breast cancer brain metastases affects brain uptake and efficacy of targeted, therapeutic nanoparticles

**DOI:** 10.1002/btm2.10108

**Published:** 2018-11-05

**Authors:** Emily A. Wyatt, Mark E. Davis

**Affiliations:** ^1^ Chemical Engineering California Institute of Technology Pasadena CA

**Keywords:** blood–brain barrier, brain metastasis, breast cancer, nanoparticle, therapeutic delivery

## Abstract

HER2‐targeted therapies effectively control systemic disease, but their efficacy against brain metastases is hindered by their low penetration of the blood‐brain and blood‐tumor barriers (BBB and BTB). We investigate brain uptake and antitumor efficacy of transferrin receptor (TfR)‐targeted, therapeutic nanoparticles designed to transcytose the BBB/BTB in three murine models. Two known models involving intracranial (IC) or intracardiac (ICD) injection of human breast cancer cells were employed, as was a third model developed here involving intravenous (IV) injection of the cells to form whole‐body tumors that eventually metastasize to the brain. We show the method of establishing brain metastases significantly affects therapeutic BBB/BTB penetration. Free drug accumulates and delays growth in IC‐ and ICD‐formed brain tumors, while non‐targeted nanoparticles show uptake and inhibition only in IC‐established metastases. TfR‐targeted nanoparticles accumulate and significantly delay growth in all three models, suggesting the IV model maintains a more intact BBB/BTB than the other models.

## 1 INTRODUCTION

Human epidermal growth factor receptor 2 (HER2) protein overexpression is observed in about 25% of human breast cancers. It confers a more aggressive phenotype and, historically, has been associated with poor patient prognosis.^1^ HER2‐targeted therapies, such as the anti‐HER2 antibody (Ab) trastuzumab, have been shown to improve outcomes in patients with HER2‐positive, metastatic disease. However, with improved control of systemic disease and prolonged survival, the incidence of brain metastases is increasing in these patients.^2^, ^3^ Currently, as many as half of patients with HER2‐positive, metastatic breast cancer develop brain metastases over time.^4^ Treatment of these brain tumors is a growing clinical challenge, in large part due to the poor penetration of HER2‐targeted agents through the blood–brain barrier (BBB).^4^, ^5^

There is considerable debate in the literature regarding the extent to which the BBB remains intact with brain metastases (in the form of the blood–tumor barrier [BTB]). In general, chemotherapy has not proven to be effective in the clinic.^6^ However, there have been a few examples of specific combinations showing clinical activity.^7^ Recent studies in experimental brain metastasis models reveal that, although the majority of metastases have some increased vascular permeability, their uptake of chemotherapeutics is limited.^8^ Furthermore, significant heterogeneity in therapeutic uptake is observed in brain metastases resected from patients, both among patients and within individual lesions.^9^ Additionally, an investigation of breast cancer subtypes showed that there is no significant disruption of the barrier by brain metastases resected from patients with HER2‐positive breast cancer.^10^ Thus, while brain metastases may have some increased permeability, approaches to overcome limited drug delivery to the brain will be important to improve clinical outcomes, particularly for HER2‐positive, metastatic disease.

Of the many strategies to increase brain penetration of systemic therapeutics, perhaps one of the most promising is the use of receptor‐mediated transcytosis (RMT).^11^, ^12^ Transferrin receptor (TfR) has been actively explored for RMT across the BBB, due to its high expression on BBB endothelium.^13^ In particular, anti‐TfR Abs has garnered the most interest because of their ability to bind TfR with high affinity without interfering with endogenous transferrin (Tf).^14^, ^15^ Results from initial studies suggested that reducing the affinity of anti‐TfR Abs to TfR maximizes their uptake into the brain parenchyma.^16^ Further investigation revealed that affinity influences intracellular trafficking; high‐affinity anti‐TfR Abs are trafficked to the lysosome, while lower‐affinity variants are more capable of transcytosis.^17^ Recently, it has been shown that bivalent Ab:TfR binding leads to lysosomal sorting, whereas monovalent binding facilitates transcytosis.^18^ In addition to affinity and valency, in vitro results suggest that pH‐sensitivity of TfR binding also affects trafficking of anti‐TfR Abs; an Ab with reduced affinity at endosomal pH 5.5 showed a greater ability to transcytose than pH‐independent Abs of comparable affinities at extracellular pH 7.4.^19^ However, despite a more detailed understanding of the properties that promote transcytosis, several challenges exist in translating anti‐TfR Abs into the clinic, including the need to: (a) dose very high quantities,^15^ (b) mitigate effector‐function driven safety concerns,^20^ and (c) develop species‐specific Abs.^21^

**Figure 1:**
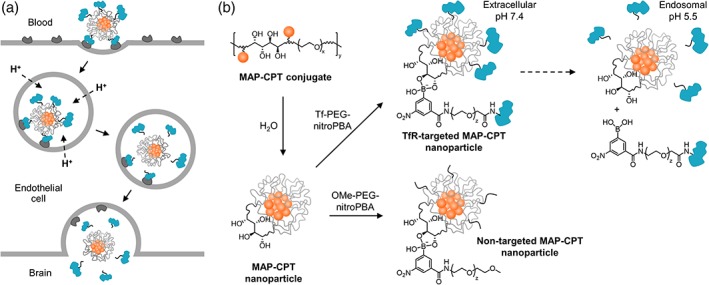
(a) Scheme of acid‐cleavable targeting strategy. Following endocytosis, rapid acidification of endosome triggers separation of Tf ligands from the nanoparticle core, allowing free diffusion of the nanoparticle into the brain parenchyma after transcytosis. (b) Preparation of TfR‐targeted and nontargeted MAP‐CPT nanoparticles and pH‐dependence of nitroPBA‐diol complex. *x* ~ 82 for 3.4 kDa PEG; *y* ~ 20 for material used in this study; *z* ~ 120 for 5 kDa PEG

Motivated by the results from anti‐TfR Ab trafficking at the BBB, we began to investigate how fundamental properties of TfR‐targeted nanoparticles affect their transcytosis capacity.^22^ Targeted nanoparticles were chosen for their ability to deliver large quantities and a variety of drugs to specific tissues at well‐controlled release rates.^23^ In analogy to the results obtained with anti‐TfR Abs, Tf‐coated gold nanoparticles (AuNPs) with reduced avidity to TfR demonstrated the greatest ability to cross the BBB.^22^ Despite showing promise, questions regarding the need for very high systemic dosing to achieve sufficient brain accumulation led to alternative nanoparticle designs. Recently, an acid‐cleavable targeting strategy was incorporated into the nanoparticle design to increase the ability of high‐avidity nanoparticles to enter the brain.^24^ With this design, nanoparticles can bind TfR with high avidity on the blood side of the BBB to enable practical, systemic dosing, but shed the targeting ligands upon acidification during transcytosis,^25^ allowing free diffusion into the parenchyma (Figure 1a). Incorporation of an acid‐cleavable linkage between Tf and the nanoparticle core increased brain uptake of high‐avidity Tf‐coated AuNPs nearly threefold.^24^ In contrast, no improvement was observed with high‐affinity anti‐TfR‐coated AuNPs with the cleavable linker, consistent with their trafficking to the lysosome. These results suggest that intracellular trafficking may also be affected by the particular targeting ligand.

Here, we determine whether nanoparticles can be prepared to deliver therapeutic quantities of drug across the BBB. We focused on HER2‐positive breast cancer brain metastasis because of the inadequate drug concentrations achieved in these tumors in the clinical setting. Although a number of preclinical models for this disease have emerged in the literature, the effect of the method used to establish metastatic brain tumors on therapeutic brain penetration has not been examined. To address these questions, we adapted a targeted nanoparticle delivery system for camptothecin (CPT) previously developed in our lab for its use at the BBB.^26^, ^27^ Tf was attached to nanoparticles consisting of a mucic acid polymer (MAP) conjugate of CPT (MAP‐CPT) through a pH‐dependent, boronic acid‐diol complexation to form TfR‐targeted MAP‐CPT nanoparticles (Figure 1b). We investigated antitumor efficacy and brain uptake of these nanoparticles in two types of models from the literature, as well as a new, third model we developed that more closely mimics the metastasis process in patients. We found that this targeted nanoparticle delivery system can be used to deliver CPT to HER2‐positive breast cancer brain metastases. Importantly, we also observed significant differences in efficacy as well as brain penetration of both TfR‐targeted and nontargeted therapeutics between the models, showing that the method of establishing brain metastases can affect brain uptake of therapeutic agents.

## 2 MATERIALS AND METHODS

Complete details of the materials and methods used in this study are provided in Supporting Information.

### 2.1 Synthesis of MAP‐CPT conjugate

Mucic acid was modified to prepare mucic acid di(Asp‐amine). Mucic acid di(Asp‐amine) was polymerized with di(succinimidyl proprionate)‐PEG to prepare MAP. Polymer molecular weight was determined by GPC. MAP was reacted with 20‐O‐Glycinylcamptothecin trifluoroacetic acid salt (CPT‐gly.TFA) to prepare MAP‐CPT conjugate. A portion of this solution was lyophilized to determine CPT content, and the remaining was formulated into 0.9% (wt/vol) saline and stored at −20 °C.

### 2.2 Synthesis of CO_2_H‐PEG‐nitroPBA and OMe‐PEG‐nitroPBA

3‐carboxy‐5‐nitrophenyl boronic acid (nitroPBA) was reacted with oxalyl chloride to prepare 3‐acyl chloride‐5‐nitrophenyl boronic acid. The acyl chloride was reacted with either CO_2_H‐PEG‐NH_2_ or OMe‐PEG‐NH_2_ to prepare CO_2_H‐PEG‐nitroPBA and OMe‐PEG‐nitroPBA, respectively.

### 2.3 Synthesis of Tf‐PEG‐nitroPBA

Tf was coupled to CO_2_H‐PEG‐nitroPBA using EDC/NHS chemistry to prepare Tf‐PEG‐nitroPBA. Protein conjugation was verified by MALDI‐TOF, using a sinapinic acid matrix.

### 2.4 Preparation of nanoparticles

Either OMe‐PEG‐nitroPBA or Tf‐PEG‐nitroPBA conjugates were added at 20x molar excess to MAP‐CPT nanoparticles to form nontargeted and TfR‐targeted nanoparticles in PBS, pH 7.4 (20 OMe or Tf per particle).

### 2.5 Nanoparticle characterization

Particle sizes and zeta potentials were measured with a Brookhaven Instruments ZetaPALS. Reported values are the average of five runs for nanoparticle size and of five runs with a target residual of 0.02 for zeta potential.

### 2.6 Nanoparticle Transwell assay

bEnd.3 cells were grown on polyester membrane transwells (Corning) until transendothelial electrical resistance was more than 30 Ohm/cm^2^. Nanoparticles were added to the apical compartment at 1 μg of CPT/well in serum‐free DMEM. The entire basal well volume was removed at 8 hr. High‐performance liquid chromatography (HPLC) was used to measure the CPT content in the basal well aliquots.

### 2.7 Antitumor efficacy in IC, ICD, and IV brain metastasis models

All animals were treated according to the NIH guidelines for animal care and use as approved by the Caltech Institutional Animal Care and Use Committee. BT474‐Gluc cells were intracranial (IC)‐, intracardiac (ICD)‐, and intravenous (IV)‐injected into female Rag2^−/−^;Il2rg^−/−^ mice, and formation of brain tumors was monitored by MRI. Mice were randomized into four groups of six mice per group: saline, CPT, nontargeted MAP‐CPT nanoparticle, and TfR‐targeted MAP‐CPT nanoparticle groups. The different formulations were freshly prepared and administered intravenously once per week for 4 weeks at a dose of 4 mg/kg (CPT basis), and tumor volume was measured weekly by MRI. For the IC model, tumor size was also monitored by measuring blood Gluc activity. Statistical significance for pairwise group comparisons was tested using the Wilcoxon‐Mann–Whitney test.

### 2.8 Measurement of CPT concentration in brain

Four mice per group were systemically administered an additional dose of each treatment at the end of the efficacy study. After 24 hr, the mice were anesthetized and perfused with PBS. Tumor and healthy brain tissue samples were collected and lysed. The CPT concentration in tissue lysate was quantified by HPLC. Statistical significance for pairwise group comparisons was tested using the Wilcoxon‐Mann–Whitney test.

### 2.9 Brain metastatic tumor cell isolation and cytotoxicity assay

BT474‐Gluc brain metastatic tumor cells were dissociated from resected brain tumors and cultured for 1 week. Sensitivity of BT474‐Gluc cells isolated from IC‐, ICD‐, and IV‐established brain tumors to CPT compared to parental cells was determined using the CellTiter 96 Aqueous One Solution cell proliferation assay (Promega), according to the manufacturer's protocol.

## 3 RESULTS AND DISCUSSION

### 3.1 Synthesis and characterization of TfR‐targeted and nontargeted MAP‐CPT nanoparticles

MAP‐CPT nanoparticles were chosen for this study because they retained the optimal design parameters identified in our previous AuNP formulations, including a sub‐100‐nm diameter and near‐neutral zeta potential.^22^ It has also been shown that these characteristics facilitate the diffusion of nanoparticles through brain tissue.^28^ The ketal linker previously investigated as the acid‐cleavable moiety between the Tf and the nanoparticle did not provide optimal cleavage kinetics to remove all surface Tf during transcytosis.^24^ The MAP delivery system allows for assembly of TfR‐targeted nanoparticles using an improved acid‐cleavable chemistry (Figure 1b), as discussed below. Furthermore, MAP‐CPT nanoparticles targeted with an antibody have already been used to effectively treat breast cancer xenografts in mice.^27^

MAP‐CPT conjugate was synthesized in a similar manner to that previously described (Supporting Information Figure S1).^26^ Properties of the material used in this study are provided in Supporting Information Table S1. MAP‐CPT conjugate was dialyzed against water to promote formation of nanoparticles with hydrophobic CPT molecules preferentially clustered in the core and vicinal diols on the surface (Figure 1b).

The boronic acid derivative, 3‐carboxy‐5‐nitrophenyl boronic acid (nitroPBA), was added to 5‐kDa polyethylene glycol (PEG), followed by conjugation of the polymer to human holo‐Tf (Supporting Information Figure S2A). A non‐targeted analog was prepared using methoxy‐terminated 5‐kDa PEG (Supporting Information Figure S2B). NitroPBA was chosen because it forms a boronic acid ester with the MAP‐CPT diols and has a pKa of 6.8.^26^ The nearly instantaneous (relative to the timeframe of BBB transcytosis) dissociation of Tf‐PEG‐nitroPBA from the nanoparticle occurs at pH <6.8, to provide ligand detachment during transcytosis.

To prepare the TfR‐targeted and nontargeted MAP‐CPT nanoparticles, either Tf‐PEG‐nitroPBA or OMe‐PEG‐nitroPBA was added to the nanoparticles at 20 M excess (Figure 1b). All nanoparticle formulations had diameters near 40 nm, as measured by dynamic light scattering, and near‐neutral zeta potentials when measured in pH 7.4 buffer (Supporting Information Table S2). The moderate increase in TfR‐targeted nanoparticle size when formulated into pH 5.5 buffer is consistent with slight steric destabilization following dissociation of Tf‐PEG‐nitroPBA conjugates from the nanoparticle surface diols at acidic pH. Importantly, no diameter increase was observed for TfR‐targeted nanoparticles after 24 hr, indicating the multi‐PEGylated Tfs in the crude Tf‐PEG‐nitroPBA mixture were not causing crosslinking between nanoparticles (Supporting Information Figure S3).

### 3.2 Specific binding of TfR allows targeted nanoparticles to cross an in vitro model of the BBB

To perform an initial screen of transcytosis capacity, we used the bEnd.3 immortalized mouse brain endothelial cell line in an established in vitro model of the BBB.^29^ Nanoparticles were added to the apical compartment of bEnd.3‐coated transwells in serum‐free DMEM and allowed to cross the model BBB for 8 hr, after which the full volume of the basal compartment was removed and CPT content measured using HPLC.

After 8 hr, TfR‐targeted MAP‐CPT nanoparticles showed a significantly increased capacity to cross the bEnd.3 cells compared to non‐targeted nanoparticles (Supporting Information Figure S4). In addition, TfR‐targeted nanoparticles showed a decreased ability to cross the model BBB when coincubated with serum concentrations of Tf, indicating TfR binding is essential to crossing. TfR‐targeted nanoparticles also showed a decreased ability to cross the transwells when coincubated with an equimolar amount of high affinity anti‐TfR Abs, but not with anti‐TfR Abs of reduced affinity at endosomal pH 5.5, consistent with previous reports of high‐affinity Ab:TfR interactions leading to lysosomal trafficking.^17^

### 3.3 Development of mouse model that replicates the metastasis process in HER2‐positive breast cancer brain metastasis patients

**Figure 2:**
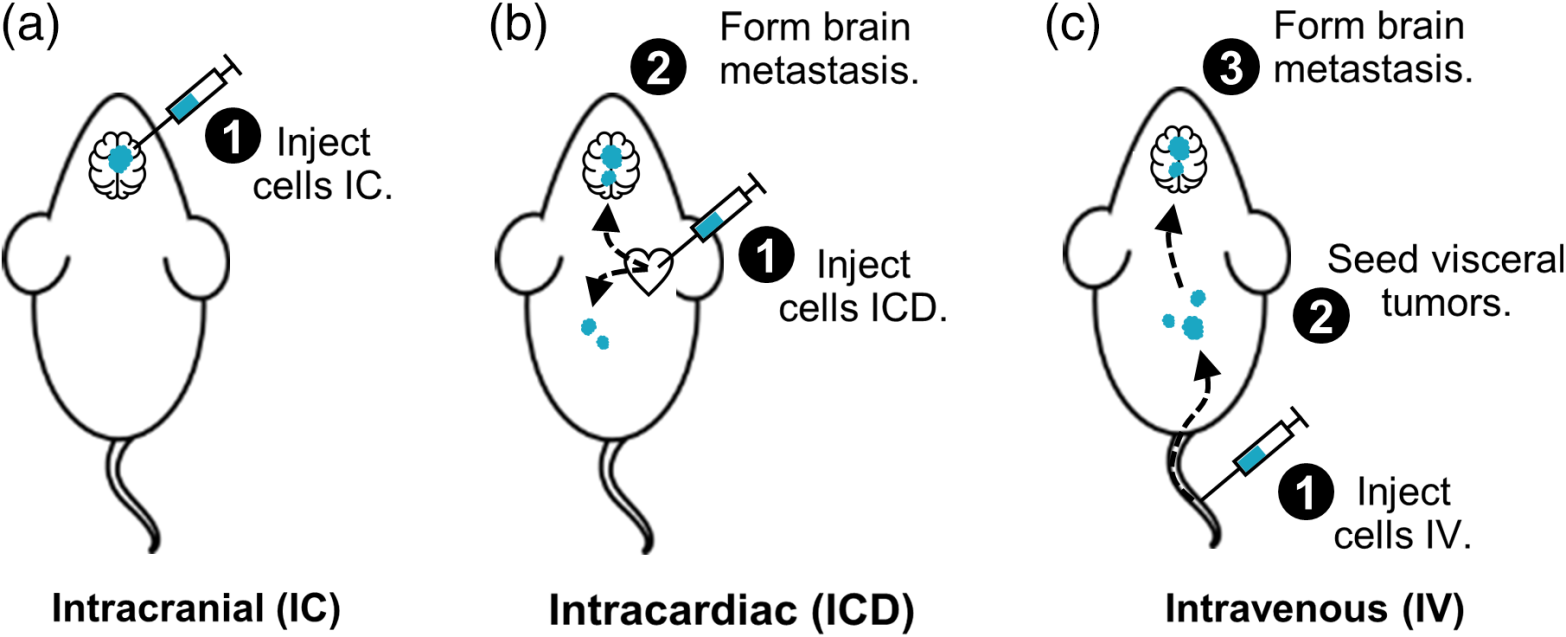
Illustration of breast cancer brain metastasis models. (a) IC injection of tumor cells allows for direct establishment of brain metastases. (b) Following ICD injection into the left ventricle, tumor cells can head to brain vasculature, as well as to other organs. Some cells will successfully extravasate and form macroscopic brain tumors. (c) After IV injection, most tumor cells will arrest in the lung capillary bed, as well as other sites, followed by subsequent metastasis to the brain

In an attempt to create a clinically representative, impermeable barrier to standard therapeutics, we developed a new model of HER2‐positive breast cancer brain metastasis that reproduces human cancer dissemination. Metastasis models are illustrated in Figure 2. HER2‐positive BT474‐Gluc cells were IV injected into Rag2^−/−^;Il2rg^−/−^ mice, and formation of brain metastases was monitored by MRI. Injection of cancer cells IV has been used to establish various metastasis models, such as lung metastasis.^30^, ^31^ This cell line was engineered to express Gaussia luciferase (Gluc) that can be used as a surrogate for tumor burden.^32^ Rag2^−/−^;Il2rg^−/−^ mice were chosen because they have shown the ability to allow multi‐organ metastatic spread of HER2‐positive breast cancer cell lines injected IV.^33^

IV injection of BT474‐Gluc cells reproduced the metastatic pattern observed in breast cancer patients, with multiple metastatic sites (Supporting Information Table S3). Importantly, brain tumors developed in a majority of the mice (>90%) before they succumbed to visceral tumor burden, with a distribution similar to that observed in patients (Supporting Information Figure S5). The median time to establishment of brain metastatic tumors visible by MRI was 4.2 months (range 2.9–6.1 months). We tested the effects of a standard anti‐HER2 therapy, trastuzumab, on the growth of BT474‐Gluc tumors established by IV injection versus the commonly used IC method. Treatment with trastuzumab led to delay in tumor progression when tumors were established by IC injection, suggesting this method of forming brain tumors may disrupt the BBB/BTB (Supporting Information Figure S6). In contrast, trastuzumab failed to control tumor growth for tumors established IV, mimicking the clinical situation.

After our development work was completed, two additional models gained popularity in the literature that use an ICD or intracarotid (ICA) injection to establish brain metastases. While the ICA model leads to the formation of fewer extracranial metastases than the ICD model, it requires additional microsurgical expertise.^34^ Because of this limitation, we chose to include the IC and ICD models from the literature in addition to our new model here to investigate the efficacy and brain penetration of TfR‐targeted MAP‐CPT nanoparticles.

### 3.4 Brain tumors show significant delay in growth with TfR‐targeted nanoparticles, but their response differs when established by different methods

We compared the efficacy of TfR‐targeted MAP‐CPT nanoparticles, nontargeted MAP‐CPT nanoparticles and CPT on the growth of BT474‐Gluc brain metastatic tumors in Rag2^−/−^;Il2rg^−/−^ mice established by IC, ICD, and IV methods (Supporting Information Figure S7). After IC, ICD, or IV injection of BT474‐Gluc cells, formation of brain metastatic tumors was monitored by MRI. Representative images and metastasis locations for each brain cancer model are provided in Supporting Information Figure S8 and Table S4, respectively. A total of six mice were used for each treatment group per model, and treatment was initiated when tumors reached 2 mm^3^ in volume. This metastasis volume was chosen as an intermediate size between small micrometastases (0.1–1 mm^3^) and large lesions (>4–10 mm^3^). The different formulations were systemically administered by lateral tail vein injection once per week for 4 weeks at a dose of 4 mg/kg (CPT basis). Brain tumor volume was measured weekly by MRI. In addition, blood Gluc activity was measured to monitor brain tumors only for the IC model, due to presence of metastases elsewhere in the mouse in the ICD and IV models.

**Figure 3:**
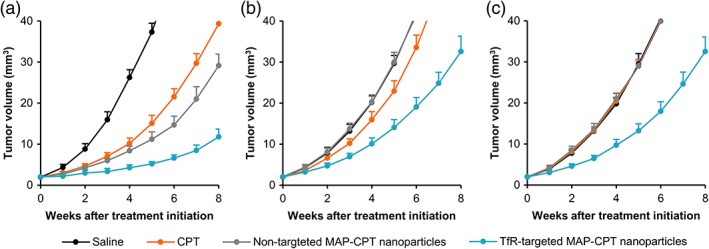
Brain tumors established using different methods show differential response to therapeutics. Tumor growth curves of BT474‐Gluc metastatic brain tumors treated with CPT (orange, 4 mg/kg), nontargeted MAP‐CPT nanoparticles (gray, 4 mg CPT/kg), and TfR‐targeted MAP‐CPT nanoparticles (blue, 4 mg CPT/kg) compared to saline (black) when established by IC (a), ICD (b), and IV injection (c). Data shown are the average of six mice per treatment group. Error bars indicate SE. *p* values for pairwise comparisons are provided in Supporting Information Tables S5–S7

In mice bearing IC‐established brain tumors, TfR‐targeted MAP‐CPT nanoparticles significantly delayed brain metastatic tumor growth compared to saline, resulting in an 8.4‐fold decrease in mean tumor volume by the end of the study (Figure 3a and Supporting Information Table S5). However, treatment with nontargeted MAP‐CPT nanoparticles or CPT also led to substantial tumor growth inhibition (3.5‐ or 2.6‐fold reduction in mean final tumor volume, respectively), supporting the hypothesis that artificial transport pathways may be introduced following IC tumor establishment. The blood Gluc activity for each treatment group correlated well with tumor volume, as measured by MRI (Supporting Information Figure S9). Individual antitumor data are provided in Supporting Information Figure S10.

In contrast to results from the IC model, only treatment with TfR‐targeted MAP‐CPT nanoparticles resulted in substantial tumor growth delay compared to saline when tumors were established by ICD injection (2.6‐fold decrease in mean tumor volume; Figure 3b and Supporting Information Table S6). Interestingly, we observed a modest response with CPT treatment, but not with nontargeted MAP‐CPT nanoparticles (although this difference was not significant).

Similar to the ICD model, with IV‐established brain tumors, TfR‐targeted MAP‐CPT nanoparticles markedly slowed tumor growth compared to saline (2.5‐fold decrease in mean tumor volume; Figure 3c and Supporting Information Table S7). Notably, no tumor growth inhibition was observed with CPT or nontargeted MAP‐CPT nanoparticles compared to saline in this model, more closely replicating the clinical situation.

### 3.5 Brain uptake of therapeutics differs in tumor, but not healthy tissue between models

To ascertain whether differences in brain penetration of the therapeutics might explain the discordance in efficacy between brain metastasis models, we systemically administered an additional dose of each treatment at the end of the efficacy study. After 24 hr, mice were anesthetized and perfused with PBS to clear any remaining nanoparticles or free drug from the bloodstream. Drug uptake into tumor and healthy brain tissue was quantified by HPLC as previously described.^27^

**Figure 4:**
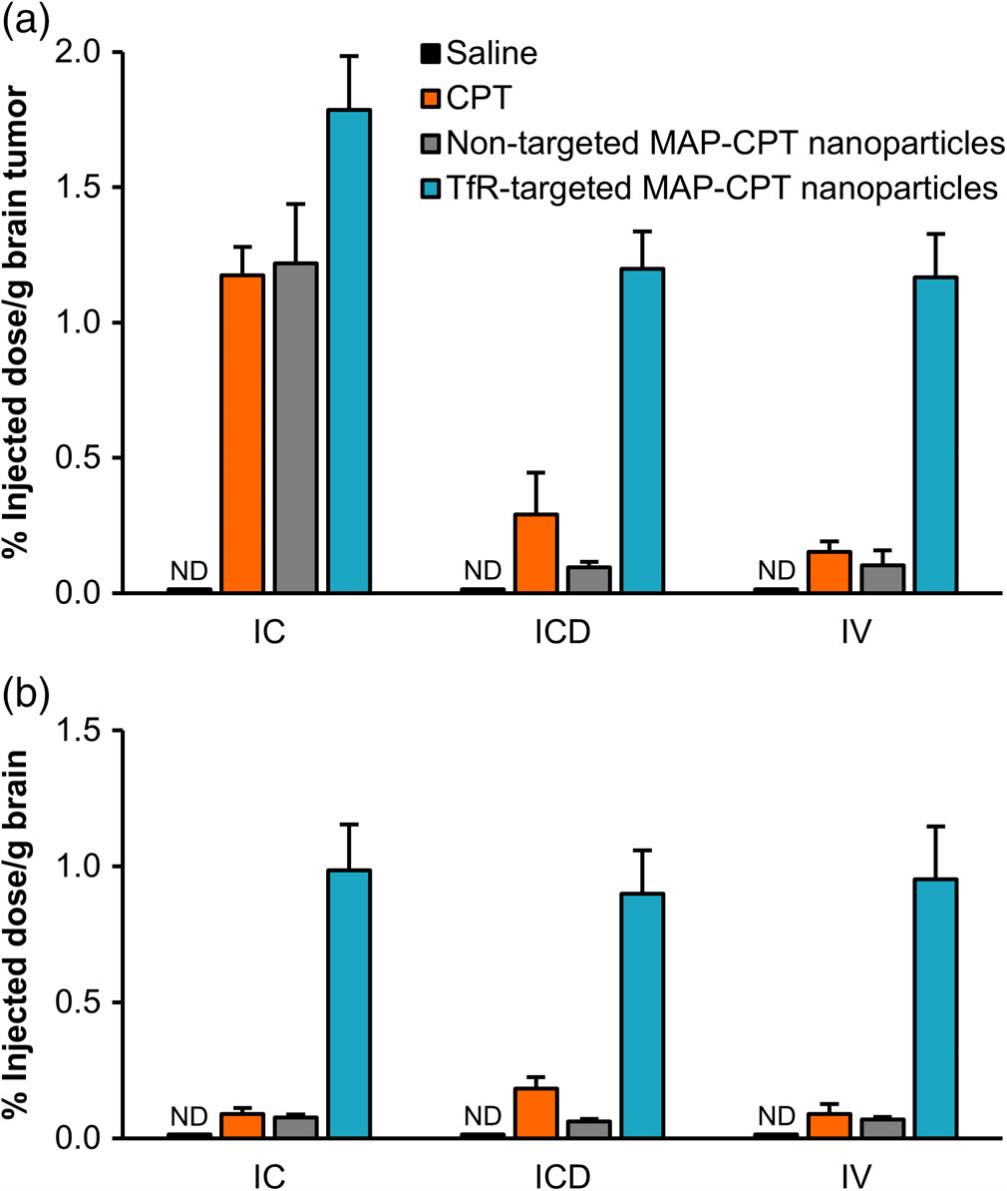
Brain uptake of therapeutics is model‐dependent in tumor, but not healthy tissue. (a) Brain uptake in BT474‐Gluc tumor tissue as calculated by percent injected dose per g of tissue for different treatments. (b) Percent injected dose in healthy brain tissue. Brain uptake was determined 24 hr after a 4 mg/kg dose (CPT basis). Data shown are the average of four mice per treatment group. Error bars indicate SE. ND, not detectable. *p* values for pairwise comparisons are provided in Supporting Information Tables S8 and S9

Tumor tissue collected from IC‐established, but not from ICD‐ and IV‐established brain tumors showed significant accumulation of CPT and nontargeted MAP‐CPT nanoparticles, consistent with the hypothesis that the barrier in IC‐established tumors may be more permeable to therapeutics than what is observed in patients with HER2‐positive disease (Figure 4a). In addition, cells isolated from BT474‐Gluc tumors from all three models as well as the respective parental cells had comparable sensitivities to CPT in vitro (Supporting Information Figure S11), ruling out permanent, model‐specific drug sensitivity as the origin for anti‐tumor differences. Although there is evidence that brain‐specific drug resistance mechanisms may also be important,^7^ these data strongly implicate BBB/BTB permeability to the therapeutic agents as a key mediator of the differential treatment response between the models in this study.

Importantly, TfR‐targeted MAP‐CPT nanoparticles showed the highest accumulation in IC‐, ICD‐, and IV‐established brain tumor tissue. In addition, TfR‐targeted nanoparticles demonstrated increased penetration into healthy brain tissue relative to free drug and nontargeted nanoparticles in all three models (Figure 4b). As with the antitumor efficacy data, these results further indicate the potential of the TfR‐targeted nanoparticle delivery system.

## 4 CONCLUSIONS

Here, we focused on understanding whether two types of breast cancer brain metastasis mouse models from the literature as well as a third, new model created in this study provide impaired drug delivery to brain metastases like what is observed for patients with HER2‐positive, metastatic breast cancer. In patients, non‐BBB‐permeable agents are unable to accumulate in brain metastases in pharmacologically active amounts. However, we did not observe this same delivery limitation in the IC model. Our results show that a non‐BBB‐penetrant small molecule (CPT) and a nontargeted nanoparticle therapeutic (ca. 30–40 nm diameter) can elicit a significant antitumor response as well as accumulate in high amounts in IC‐established brain tumors. In contrast to the IC model, both the ICD and IV models provide for a more intact BBB/BTB. Our results indicate that the ICD model may allow for a slightly increased permeability to small molecule drugs, but not to larger nanoparticle entities when compared to the IV model. Consistent with a modest uptake in healthy brain tissue, it is possible that the high number of microscopic tumor foci commonly observed throughout the brain following ICD injection may contribute to a slight net increase in parenchymal penetration as a whole. Nevertheless, this effect was minimal.

Most importantly, our data show that the method of establishing brain tumors can dramatically affect the efficacy of therapeutics and their brain penetration. Our findings suggest that, although the IC model allows for consistent and reproducible tumor growth in the brain parenchyma and thus may be useful for studying basic biological mechanisms, this model must be used with caution for translational research with diseases where a nonpermissive BBB is clinically relevant. While tumor burden is not as consistent in the ICD and IV models, our data support the use of these models if the experimenter is interested in transport properties of a given therapeutic.

Additionally, we show that TfR‐targeted nanoparticles are capable of delivering a small molecule chemotherapeutic, CPT, to HER2‐positive breast cancer brain metastases. We observed that TfR‐targeted MAP‐CPT nanoparticles significantly slowed tumor growth in the brain and demonstrated increased accumulation in brain metastases relative to free drug and nontargeted nanoparticles. The specific example of assembling a TfR‐targeted nanoparticle system for CPT was selected to test the delivery strategy. CPT is not a particularly good drug for use with BT474 cells (relative to other breast cancer cell lines).^26^ Thus, it is encouraging to observe tumor growth delay when delivering CPT via targeted nanoparticles to the BT474‐Gluc brain metastases. It is expected that TfR‐targeted nanoparticles delivering therapeutic agents with greater potency will reveal even more significant tumor size reductions.

Further, it is important to note that TfR‐targeted nanoparticles accumulated in significant amounts in healthy brain tissue when compared to free drug and nontargeted nanoparticles in all three models. This observed whole‐brain penetration has implications for the selection of therapeutics that should be incorporated into this delivery system and of target diseases. In the case of brain cancers, the ability to penetrate not only tumor tissue, but also healthy tissue could be advantageous in accessing micrometastases or fingers of glioma tumors that are frequently the reason for treatment failure. At the same time, the broad nanoparticle accumulation in the brain will require careful thought as to which drugs are used in this application, due to potential toxicity issues. For other brain diseases where whole‐brain therapeutic exposure is highly desired, such as neurodegenerative diseases, this targeted nanoparticle system may offer a compelling approach to delivering therapeutics across an intact BBB.

Here, we show that the method used to establish breast cancer brain metastases can affect efficacy and brain uptake of therapeutic agents. We observed a significant antitumor response as well as brain tumor accumulation of a non‐BBB‐penetrant small molecule and a nontargeted nanoparticle therapeutic in tumors that were formed by IC injection of human breast cancer cells. In contrast, both ICD and IV injection of the cancer cells provided for a more clinically relevant, impermeable BBB/BTB to nonpenetrant agents. Additionally, we show that TfR‐targeted MAP‐CPT nanoparticles can accumulate in brain metastases in greater amounts and lead to improved antitumor activity compared with free drug and nontargeted MAP‐CPT nanoparticles. Furthermore, TfR‐targeted nanoparticles showed an increased ability to cross an intact BBB, resulting in whole‐brain therapeutic accumulation.

## AUTHOR CONTRIBUTIONS

E.A.W. and M.E.D. designed research; E.A.W. performed research; E.A.W. and M.E.D. analyzed data; and E.A.W. and M.E.D. wrote the paper.

## Supporting information

Supporting Information
