# Supplementary material for: Method of establishing breast cancer brain metastases affects brain uptake and efficacy of targeted, therapeutic nanoparticles
Source: Bioeng Transl Med. 2018 Nov 5;4(1):30–7. doi: 10.1002/btm2.10108 (PMC6336738; doi:10.1002/btm2.10108)
Supplement: Supplementary file 1 — Supporting Information [file BTM2-4-30-s001.docx]

**Supporting Information**

**Materials and Methods**

**Synthesis of MAP-CPT Conjugate.**

^1^H NMR spectra were acquired on a Varian 600 MHz spectrometer (Inova). Electrospray ionization (ESI) masses of small molecules were acquired on a Finnigan LCQ ion trap mass spectrometer. Matrix-assisted laser desorption/ionization-time-of-flight (MALDI-TOF) mass spectra for polymers were acquired on an Applied Biosystems Voyager DE-PRO.

***Synthesis of Mucic Acid Dimethyl Ester.*** Methanol (360 mL) was added to mucic acid (15 g, 1 equiv, Alfa Aesar) in a 500 mL round-bottomed flask. To this was added concentrated sulfuric acid (1.2 mL, 0.3 equiv). The suspension was stirred and refluxed at 85 ºC overnight. The mixture was cooled to room temperature and filtered through a Buchner funnel using Whatman Grade 5 filter paper. The solid was washed with methanol (600 mL), and recrystallized with a mixture of methanol (240 mL) and triethylamine (1.5 mL) at 85 ºC for 1 h. The mixture was again cooled to room temperature and filtered. The solid was washed with methanol (600 mL), and dried under vacuum at 75 ºC overnight to yield mucic acid dimethyl ester (14.2 g) as a white solid. ^1^H NMR (600 MHz, DMSO-*d*_6_): 4.91 (d, 2H), 4.80 (q, 2H), 4.28 (d, 2H), 3.78 (q, 2H), 3.63 (s, 6H). ESI/MS: 261.0 [M+Na]^+^.

***Synthesis of N-Boc-Protected Mucic Acid Ethylenediamine.*** Methanol (225 mL) was added to mucic acid dimethyl ester (14.2 g, 1 equiv) in a 500 mL round-bottomed flask. To this was added triethylamine (21.7 mL, 2.6 equiv), and the mixture was stirred and refluxed at 85 ºC for 30 min, forming a yellow suspension. N-Boc-ethylenediamine (24.6 mL, 2.6 equiv, AK Scientific) in methanol (55 mL) was added, and the reaction was stirred and refluxed at 85 ºC overnight. The mixture was cooled to room temperature, and filtered through a Buchner funnel using Whatman Grade 5 filter paper. The solid was washed with methanol (750 mL), and recrystallized with methanol (350 mL) at 85 ºC for 1.5 h. The mixture was again cooled to room temperature and filtered. The solid was washed with methanol (750 mL), and dried under vacuum at 75 ºC overnight to yield N-Boc-protected mucic acid ethylenediamine (19.2 g) as a white solid. ^1^H NMR (600 MHz, DMSO-*d*_6_): 7.71 (t, 2H), 6.81 (t, 2H), 5.13 (d, 2H), 4.35 (q, 2H), 4.09 (d, 2H), 3.77 (q, 2H), 3.12 (m, 4H), 2.98 (m, 4H), 1.36 (s, 18). ESI/MS: 517.1 [M+Na]^+^.

***Synthesis of Mucic Acid Ethylenediamine.*** N-Boc-protected mucic acid ethylenediamine (19.2 g) in a 500 mL round-bottomed flask was placed in a water bath. 3 N hydrochloric acid in methanol (325 mL) was added, and the reaction flask was sealed and vented with a needle. The suspension was stirred at 25 ºC for 8 h. The slurry was filtered through a glass frit with a fine grain, and washed with methanol (900 mL) until the filtrate pH was close to neutral. The solid was dried under vacuum at 80 ºC overnight to yield mucic acid ethylenediamine (11.5 g) as a white solid. ^1^H NMR (600 MHz, DMSO-*d*_6_): 7.97–7.84 (m, 8H), 5.30 (d, 2H), 4.58 (d, 2H), 4.16 (d, 2H), 3.82 (m, 2H), 3.39–3.32 (m, 4H), 2.85 (m, 4H). ESI/MS: 295.0 [M+H]^+^.

***Synthesis of Mucic Acid Di(Asp(OBzl)-Boc).*** Mucic acid ethylenediamine (3 g, 1 equiv) was dissolved in 30 mL DMSO in a 250 mL round-bottomed flask. To this was added Boc-Asp(OBzl)-OSu (10.3 g, 3 equiv, Bachem) in acetonitrile (80 mL) and pyridine (3.2 mL, 5 equiv). The reaction was stirred and refluxed at 60 ºC overnight. The mixture was cooled to room temperature, and acetonitrile was removed by rotary evaporation. The solution was precipitated by addition of nanopure water, and the precipitate was recrystallized with nanopure water (100 mL) at 85 ºC for 1 h. The mixture was cooled to room temperature, filtered through a glass frit with a fine grain, and washed with nanopure water (200 mL). The recrystallization procedure was repeated with acetonitrile. The solid was dried under vacuum at 50 ºC overnight to yield mucic acid di(Asp(OBzl)-Boc) (2.1 g) as a white solid. ^1^H NMR (600 MHz, DMSO-*d*_6_): 7.94 (t, 2H), 7.76 (t, 2H), 7.37–7.31 (m, 10H), 7.06 (d, 2H), 5.13–5.08 (m, 6H), 4.37–4.32 (d, 2H), 4.30–4.28 (d, 2H), 4.14–4.12 (d, 2H), 3.81–3.79 (d, 2H), 3.18–3.09 (m, 8H), 2.79–2.57 (m, 4H), 1.38 (s, 18H). ESI/MS: 905.0 [M+H]^+^.

***Synthesis of Mucic Acid Di(Asp(OBzl)-amine).*** Dichloromethane (18 mL) was added to mucic acid di(Asp(OBzl)-Boc) (2.1 g, 1 equiv) in a 50 mL round-bottomed flask vented with argon. The flask was cooled to 0 ºC in an ice bath, and trifluoroacetic acid (6 mL, 36 equiv) was added dropwise. The reaction was stirred for 8 h under argon, slowly equilibrating to room temperature. Solvent was removed by rotary evaporation. The solid was dissolved in dichloromethane (30 mL) and dried by rotary evaporation twice more, and then recrystallized with tetrahydrofuran (30 mL) at 55 ºC for 1 h. The mixture was cooled to room temperature and filtered through a glass frit with a fine grain. The solid was washed with tetrahydrofuran (100 mL), and dried under vacuum at 50 ºC overnight to yield mucic acid di(Asp(OBzl)-amine) (1.4 g) as a white solid. ^1^H NMR (600 MHz, DMSO-*d*_6_): 8.46 (t, 2H), 8.21 (s, 6H), 7.80 (t, 2H), 7.39–7.35 (m, 10H), 5.19–5.16 (t, 2H), 5.13 (s, 4H), 4.41 (s, 2H), 4.15–4.13 (d, 2H), 4.06–4.04 (d, 2H), 3.83 (s, 2H), 3.22–3.16 (m, 8H), 3.02–2.83 (m, 4H). ESI/MS: 705.3 [M+H]^+^.

***Synthesis of Mucic Acid Di(Asp-amine).*** Methanol (50 mL) was added to mucic acid di(Asp(OBzl)-amine) (1.4 g, 1 equiv) and 20% (w) palladium hydroxide on carbon (568 mg, 10 equiv) in a 100 mL round-bottomed flask. The reaction flask was sealed and vented with argon for 30 min. Hydrogen gas was added by a double-layered balloon, and the reaction was stirred for 24 h at room temperature. Catalyst was separated by centrifugation at 3220 g for 15 min, and the solvent removed by rotary evaporation. The solid was reconstituted in nanopure water, and the solution was filtered through a 0.2 µm Supor membrane Acrodisc syringe filter (Pall) and lyophilized to yield mucic acid di(Asp-amine) (1.1 g) as a white solid. ^1^H NMR (600 MHz, DMSO-*d*_6_): 8.39 (t, 2H), 8.18 (broad, 6H), 7.77 (t, 2H), 5.18 (t, 2H), 4.46 (s, 2H), 4.12 (s, 2H), 3.96–3.94 (m, 2H), 3.79 (s, 2H), 3.21–3.11 (m, 8H), 2.84–2.65 (m, 4H). ESI/MS: 525.2 [M+H]^+^.

The product was stored under argon at -20 ºC.

***Synthesis of Mucic Acid Polymer (MAP).*** Mucic acid di(Asp-amine) (220 mg, 1 equiv) and di(succinimidyl proprionate)-PEG (3.4 kDa, 1 g, 1 equiv, JenKem) were equilibrated to room temperature for 1 h, then added to an oven-dried 10 mL round-bottomed flask. The reaction flask was sealed, and the two solids were dried under vacuum for 4 h. Anhydrous dimethyl sulfoxide (7 mL) was added under argon to dissolve the two solids. To this was added anhydrous N,N-diisopropylethylamine (205 µL, 4 equiv) dried over molecular sieves, and the solution was stirred under argon at room temperature for 42 h. The solution was dialyzed against dimethyl sulfoxide and nanopure water using a 10 kDa MWCO Spectra/Por 7 membrane (Spectrum), filtered through a 0.2 µm Supor membrane Acrodisc syringe filter (Pall) and lyophilized to yield MAP (983 mg) as a white, sponge-like solid. ^1^H NMR (600 MHz, DMSO-*d*_6_): 8.11 (d, 1H), 8.08 (d, 1H), 7.83 (t, 1H), 7.79 (t, 1H), 7.73 (t, 2H), 4.49 (td, 2H), 4.14 (d, 2H), 3.69 (ddt, 2H), 3.59 (t, 4.3H), 3.53–3.43 (s - PEG), 3.18–3.07 (m, 8H), 2.61–2.43 (m, 4H), 2.38 (t, 4.3H).

***Determination of MAP Molecular Weight.*** Polymer molecular weight was determined on a gel permeation chromatography (GPC) system equipped with an Agilent 1100 HPLC with binary pump and injector with 2 size exclusion columns in series (PL aquagel-OH 40 8 µm, Agilent) connected to Wyatt DAWN HELEOS light scattering and Wyatt Optilab rEX refractive index detectors. MAP was dissolved at six different concentrations in PBS, pH 7.4 and directly injected into the refractive index detector at 0.2 mL/min using a syringe pump to determine specific refractive increment, d*n*/dc. Absolute molecular weight was determined by injecting 100 µL of MAP dissolved at 4 mg/mL in PBS, pH 7.4 onto the column. PBS was used as the eluent at a flow rate of 0.7 mL/min, and the detected polymer peak was analyzed using ASTRA V Software.

***Synthesis of MAP-CPT Conjugate.*** Anhydrous dimethyl sulfoxide (10 mL) was added under argon to dissolve MAP (200 mg, 1 equiv) in a 25 mL round-bottomed flask. To this was added EDC (83 mg, 4 equiv) and NHS (32 mg, 3 equiv) dissolved in anhydrous dimethyl sulfoxide (3 mL), followed by 20-O-Glycincamptothecin trifluoroacetic acid salt (CPT-gly.TFA, 170 mg, 3 equiv) dissolved in dimethyl sulfoxide (3 mL) and anhydrous N,N-diisopropylethylamine (56 µL) dried over molecular sieves. The reaction was stirred under argon at room temperature overnight. The solution was dialyzed against dimethyl sulfoxide 3 times and nanopure water 2 times using a 10 kDa MWCO Spectra/Por 7 membrane (Spectrum). Precipitate was removed by centrifugation at 3220 g for 15 min, and the supernatant was filtered through a 0.2 µm Supor membrane Acrodisc syringe filter (Pall) to yield MAP-CPT conjugate as self-assembled nanoparticles in solution. A portion of this clear yellow solution was lyophilized to determine percent CPT conjugation. The remaining product was formulated into 0.9% (w/v) saline and stored at -20 ºC.

***Determination of CPT Content in MAP-CPT.*** Lyophilized MAP-CPT was dissolved in dimethyl sulfoxide at 10 mg/mL, diluted to 0.1 mg/mL with 1 N NaOH, and incubated overnight. Fluorescence was measured at 370/440 nm (ex/em) using a Safire 2 multi-mode plate reader (Tecan). A calibration curve of known concentrations of CPT was prepared and used to determine the CPT concentration in the mixture.

**Synthesis of CO_2_H-PEG-nitroPBA and OMe-PEG-nitroPBA.**

***Synthesis of 3-acyl chloride-5-nitrophenyl boronic acid.*** 3-carboxy-5-nitrophenyl boronic acid (nitroPBA, 100 mg, 1 equiv, Alfa Aesar) was added to an oven-dried 10 mL round-bottomed flask. The reaction flask was sealed and vented with argon. Anhydrous tetrahydrofuran with BHT inhibitor (4 mL) was added to dissolve the boronic acid, followed by anhydrous dimethylformamide (7 µL, 0.2 equiv). The flask was cooled to 0 ºC in an ice bath, and oxalyl chloride (98 µL, 2.4 equiv) was added dropwise. After addition of oxalyl chloride, the ice bath was removed and the reaction was stirred under argon for 2 hrs. Solvent was evaporated under vacuum to yield 3-acyl chloride-5-nitrophenyl boronic acid (108 mg) as a yellow solid.

***Synthesis of CO_2_H-PEG-nitroPBA and OMe-PEG-nitroPBA.*** 3-acyl chloride-5-nitrophenyl boronic acid (46 mg, 2 equiv) was added to an oven-dried 25 mL round-bottomed flask. The reaction flask was sealed, vented with argon, and cooled to 0 ºC in an ice bath. Anhydrous DCM (5 mL) was added to dissolve the boronic acid. Acetic acid-PEG-amine (5 kDa, 500 mg, 1 equiv, JenKem) was added to a separate oven-dried 10 mL round-bottomed flask. The flask was sealed, and vented with argon. To this was added anhydrous N,N-diisopropylethylamine (35 µL, 2 equiv) dried over molecular sieves, and anhydrous DCM (5 mL) to dissolve the PEG. The PEG solution was added dropwise to the boronic acid solution. The reaction flask was left in the ice bath to slowly warm to room temperature, and stirred under argon overnight protected from light. Solvent was removed under vacuum, and the solid reconstituted in 0.5 N HCl (5 mL) and stirred for 15 min. The solution was filtered through a 0.2 µm Supor membrane Acrodisc syringe filter (Pall) and dialyzed against nanopure water until constant pH using a 15 mL Amicon Ultra 3 kDa spin filter (EMD Millipore), and lyophilized to yield CO_2_H-PEG-nitroPBA (465 mg) as a white solid. ^1^H NMR (600 MHz, DMSO-*d*_6_): 12.52 (s - COOH, 1H), 8.90 (t, 1H), 8.73 (m, 1H), 8.69 (m, 1H), 8.65 (m, 1H), 8.61 (s, 2H), 4.00 (s, 2H), 3.53–3.46 (s - PEG). MALDI: 5496.0.

A similar procedure was followed using methoxy-PEG-amine (5 kDa, 500 mg, 1 equiv, JenKem) to synthesize OMe-PEG-nitroPBA. ^1^H NMR (600 MHz, DMSO-*d*_6_): 8.90 (t, 1H), 8.72 (m, 1H), 8.69 (m, 1H), 8.64 (m, 1H), 8.60 (s, 2H), 3.54–3.48 (s - PEG), 3.23 (s, 2H). MALDI: 5825.4.

**Synthesis of Tf-PEG-nitroPBA.** CO_2_H-PEG-nitroPBA (16 mg, 25 equiv), EDC-HCl (6.1 mg, 250 equiv), and NHS (5.5 mg, 375 equiv) were dissolved in 0.1 M MES buffer, pH 6.0 (0.33 mL), and stirred for 15 min at room temperature. The reaction mixture was then added to a 0.5 mL Amicon Ultra 3 kDa spin filter (EMD Millipore), and centrifuged to isolate the activated nitroPBA-PEG-NHS ester. The ester was added to human holo-Tf (10 mg, 1 equiv, Sigma) dissolved in 0.1 M PBS, 0.15 M NaCl, pH 7.4 (1 mL). The reaction was lightly agitated for 2 h at room temperature, and then dialyzed against 0.1 M PBS, 0.15 M NaCl, pH 7.4 using 0.5 mL Amicon Ultra 50 kDa spin filters (EMD Millipore) to remove excess PEG. A portion of this solution was dialyzed into 10 mM PB, pH 7.4, and conjugation was verified by MALDI-TOF (autoflex speed TOF/TOF, Bruker) using a sinapinic acid matrix. MALDI-TOF: 85295.4. The amount of iron loaded to the Tf was verified by UV-VIS on a NanoDrop system (Thermo Scientific) using the ratio of A_465_/A_280_. This ratio was compared to that of the unreacted human holo-Tf, and a value ≥ 80% of the unreacted ratio confirmed adequate iron retention following synthesis steps. The remaining Tf-PEG-nitroPBA was formulated into PBS, pH 7.4, and stored at 4 ºC.

**Preparation of Nanoparticles.** Either OMe-PEG-nitroPBA or Tf-PEG-nitroPBA conjugates in PBS, pH 7.4 were added at 20x molar excess to MAP-CPT nanoparticles to form non-targeted and TfR-targeted MAP-CPT nanoparticles, respectively (20 OMe or Tf per particle). The solution was gently mixed by pipette, and allowed to equilibrate for 10 min. Nanoparticle formulations were filtered using a 0.45 µm PTFE membrane Millex-LH syringe filter (EMD Millipore).

**Nanoparticle Characterization.** Nanoparticles were characterized using a Brookhaven Instruments Corporation (BIC) ZetaPALS. Nanoparticles were diluted in PBS, pH 7.4 or PB, pH 5.5, and hydrodynamic diameter was measured by dynamic light scattering (DLS) using BIC Particle Sizing Software. Measurements were performed in solutions of different pH to allow for characterization under conditions where OMe-PEG-nitroPBA and Tf-PEG-nitroPBA conjugates would be bound to the vicinal diols on MAP (pH 7.4) and where nitroPBA conjugates would dissociate from the diols on MAP (pH 5.5). Particle formulations were diluted in 10 mM PB, pH 7.4 or 1 mM KCl, pH 5.5, and zeta potential was measured using BIC PALS Zeta Potential Analyzer software with a target residual of 0.02. Five runs were performed for both the nanoparticle diameter and zeta potential measurements.

**Nanoparticle Transwell Assay.** bEnd.3 cells were obtained from ATCC and maintained in Dulbecco’s modified Eagle’s medium (DMEM) supplemented with 10% (v/v) FBS and 1% penicillin/streptomycin in a humidified oven at 37 °C with 5% CO_2_. Media was added to apical and basal wells of 12 mm polyester-coated Transwell supports (Corning), and allowed to equilibrate overnight at 37 ºC. Cells were added to the apical well at 82,500 cells/well. Media was replaced in the apical and basal wells every 2 days. Transepithelial electrical resistance (TEER) was measured in an Endohm chamber using an EVOM resistance meter (World Precision Instruments). Once TEER reached ≥ 30 Ohm∙cm^2^, transcytosis experiments were performed. Prior to introduction, both compartments of the Transwell were washed with serum-free DMEM, and allowed to equilibrate for 1 h. Nanoparticles were added at 1 µg of CPT/well to the apical well. After 8 h, the entire volume was removed from the basal well, and immediately frozen at -80 ºC until time for analysis. For the Tf-competition assay, the experiment was performed as described above using DMEM + 2.5 mg/mL Tf as the media in both apical and basal wells. For the high affinity anti-TfR Ab coincubation assay, the nanoparticles were formulated with an equimolar (Tf:Ab) amount of R17217 anti-TfR Ab (Biolegend) in serum-free DMEM and added to the apical chamber. For the pH-dependent anti-TfR Ab coincubation assay, the nanoparticles were formulated with an equimolar (Tf:Ab) amount of MEM-189 anti-TfR Ab (Thermo Fisher) in serum-free DMEM and added to the apical chamber.

The amount of CPT in the basal well was determined on an Agilent 1100 HPLC system with a reverse phase column (Synergi 4 µm Hydro-RP 80 Å, Phenomenex) connected to a fluorescence detector set to 370/440 nm (ex/em). 50% acetonitrile/50% potassium phosphate buffer (10 mM, pH 4) was used as the eluent at a flow rate of 0.5 mL/min. To cleave CPT from the MAP polymer, 13 µL of 0.1 N NaOH was added to 20 µL sample and incubated for 1 h. Then, 20 µL of 0.2 N HCl was added to convert the carboxylate CPT to the lactone form, followed by 30 min incubation. Subsequently, 147 µL methanol was added, and the mixture incubated for 2 h at room temperature for protein precipitation. The sample was centrifuged at 14000 g for 15 min and supernatant filtered using a 0.45 µm PTFE membrane Millex-LH syringe filter (EMD Millipore). CPT content was determined by injecting 100 µL of the filtered solution onto the column compared to a calibration curve of known concentrations of CPT. Reported values are the average of four wells per group. The error shown is standard error of the mean. Pairwise group comparisons testing for statistically significant differences were performed using the Wilcoxon-Mann-Whitney test in MATLAB.

**Antitumor Efficacy in IC, ICD, and IV Brain Metastasis Models.**

***IC, ICD and IV Tumor Models.*** All animals were treated according to the NIH guidelines for animal care and use as approved by the Caltech Institutional Animal Care and Use Committee. BT474-Gluc cells, transduced with an expression cassette encoding Gluc and CFP separated by an internal ribosomal entry site using a lentiviral vector, were obtained from Dr. Jain at Harvard University. BT474-Gluc cells were maintained in RPMI 1640 supplemented with 10% (v/v) FBS in a humidified oven at 37 °C with 5% CO_2_. For the IC model, 50,000 BT474-Gluc cells in 2 µL RPMI were intracranially injected into the right cerebral hemisphere of female Rag2^-/-^;Il2rg^-/-^ mice (Jackson Laboratory) using a stereotaxic apparatus at a rate of 0.1 µL/min. The coordinates for injection were 2 mm posterior, 1.5 mm lateral to bregma, and 2.5 mm depth from bregma. For the ICD model, 100,000 BT474-Gluc cells were suspended in 100 µL of RPMI and slowly injected into the left ventricle of female Rag2^-/-^;Il2rg^-/-^ mice. Injections were performed blind, midway between the sternal notch and top of xyphoid process, and 13% anatomical left of sternum. Successful insertion into the left cardiac ventricle was confirmed by a bright red pulse of blood in the syringe. For the IV model, 2 M cells were suspended in 150 µL RPMI and slowly injected into the lateral tail vein of restrained female Rag2^-/-^;Il2rg^-/-^ mice.

***Tumor Size Monitoring.*** For the IC model, tumors formed at the site of injection were monitored weekly by MRI on a 11.7 T magnet. For ICD and IV models, formation of BT474-Gluc brain metastatic tumors was monitored by MRI every third week until macroscopic tumors were visible (~0.2 mm^3^ in volume). Tumor growth was then monitored weekly by MRI, as for the IC model. Mice were anaesthetized with 1.5–2% (v/v) isoflurane in O_2_ at a flow rate of 1–1.5 mL/min. T2-weighted 3D RARE images were acquired to assess the tumor volume. The image acquisition parameters were as follows: echo time: 6.1 ms; repetition time: 250 ms; rapid acquisition relaxation enhanced (RARE) factor: 4; number of averages: 4; field of view: 2.0 cm x 1.2 cm x 0.8 cm; matrix: 200 x 120 x 80 (100 µm isotropic resolution). Tumor volume was determined manually from the T2 hyperintense tumor regions of the brain using Fiji software. For the IC model, tumor size was also monitored by measuring the activity of secreted Gluc in the blood. 20 µL of blood was collected weekly from the saphenous vein, mixed with 5 µL of 50 mM EDTA, and immediately frozen at -20 ºC until time for analysis. Blood was transferred to an opaque 96-well plate (Nunc), and Gluc activity measured using the Pierce Gaussia Luciferase Flash Assay Kit, according to the manufacturer’s protocol. Photon counts were acquired for 5 s following addition of coelenterazine using a Safire 2 multi-mode plate reader (Tecan). Pairwise group comparisons testing for statistically significant differences were performed using the Wilcoxon-Mann-Whitney test in MATLAB.

***Treatments.*** Treatment began when brain metastatic tumors reached ~2 mm^3^, as measured by MRI. Mice in each model were randomized into four groups of six mice per group. CPT, non-targeted MAP-CPT nanoparticles, and TfR-targeted MAP-CPT nanoparticles were freshly prepared. The different formulations were systemically administered by lateral tail vein injection once per week for 4 weeks at a dose of 4 mg/kg (CPT basis). Injections were standardized to 150 µL per 20 g body weight. CPT is highly insoluble in aqueous solutions; therefore, it was dissolved in a solution containing 20% DMSO, 20% PEG 400, 30% ethanol, and 30% 10 mM pH 3.5 phosphoric acid. Nanoparticle treatments were prepared and administered in PBS, pH 7.4 as previously described. The control treatment was 0.9% (w/v) saline.

No gross signs of toxicity were observed from either the non-targeted or the targeted nanoparticles in our study, while animals did have reactions to dosing with the CPT alone. These reactions are common and documented in the literature for CPT (1,2).

**Measurement of CPT Concentration in Brain.** Eight weeks after the beginning of the treatment, four of six mice per treatment group were systemically administered by lateral tail vein injection one additional dose at 4 mg/kg (CPT basis). After 24 h, the mice were anaesthetized and transcardially perfused with PBS, pH 7.4. Brain tumors were collected and sectioned into two approximately equal sized pieces. An equally sized piece of healthy brain tissue was collected from the brain region contralateral to the tumor location. One piece of the tumor and the healthy tissue were weighed and placed in separate Lysing Matrix A tubes containing ¼ inch ceramic spheres (MP Biomedicals) in RIPA buffer (Cell Signaling Technologies) at a fixed ratio (w/v). Tissues were homogenized using a FastPrep-24 homogenizer (MP Biomedicals) at a rate of 6 m/s for 30 s. A total of three homogenization steps occurred with a 1 min rest on ice between steps. After the final homogenization step, samples were rotated for 30 min, then centrifuged at 14000 g for 15 min at 4 ºC. The supernatant was collected, and immediately frozen at -80 ºC until time for analysis. CPT uptake into tumor and healthy brain tissue for each formulation was determined by HPLC, as described above. Pairwise group comparisons testing for statistically significant differences were performed using the Wilcoxon-Mann-Whitney test in MATLAB.

**Brain Metastatic Tumor Cell Isolation and Cytotoxicity Assay.** The second piece of brain tumor tissue was immediately minced ice cold RPMI, and incubated in RPMI supplemented with 10% (v/v) FBS, 1% penicillin/streptomycin and 1 mg/mL collagenase/dispase enzyme mix (Roche) at 37 °C for 1 h with shaking. The tissue was then centrifuged at 300 g for 5 min, and supernatant removed. The pellet was resuspended in RPMI supplemented with 10% (v/v) FBS and 1% penicillin/streptomycin, and the cells cultured in a humidified oven at 37 °C with 5% CO_2_. Media was refreshed after 24 h, and every 2 days thereafter. After 1 week, the majority of the cells were BT474-Gluc tumor cells, as identified by CFP.

BT474-Gluc cells dissociated from the brain parenchyma, as well as parental cells, were seeded at a density of 3,000 cells/well in 96-well plates. After 24 h, media was removed and replaced with fresh media containing different concentrations of CPT. After 72 h, BT474-Gluc cell viability was measured using the CellTiter 96 Aqueous One Solution cell proliferation assay (Promega) on an Infinite M200 microplate reader (Tecan), according to the manufacturer’s protocol.

**References**

1. Moertel CG, Schutt AJ, Reitemeier RJ, Hahn RG (1972) Phase II study of camptothecin (NSC-100880) in the treatment of advanced gastrointestinal cancer. *Cancer Chemotherapy Reports* 56(1):95–101.
2. Muggia FM, Creaven PJ, Hansen HH, Cohen MH, Selawry OS (1972) Phase I clinical trial of weekly and daily treatment with camptothecin (NSC-100880): correlation with preclinical studies. *Cancer Chemotherapy Reports* 56(4):515–521.

**Supplemental Figures**

**
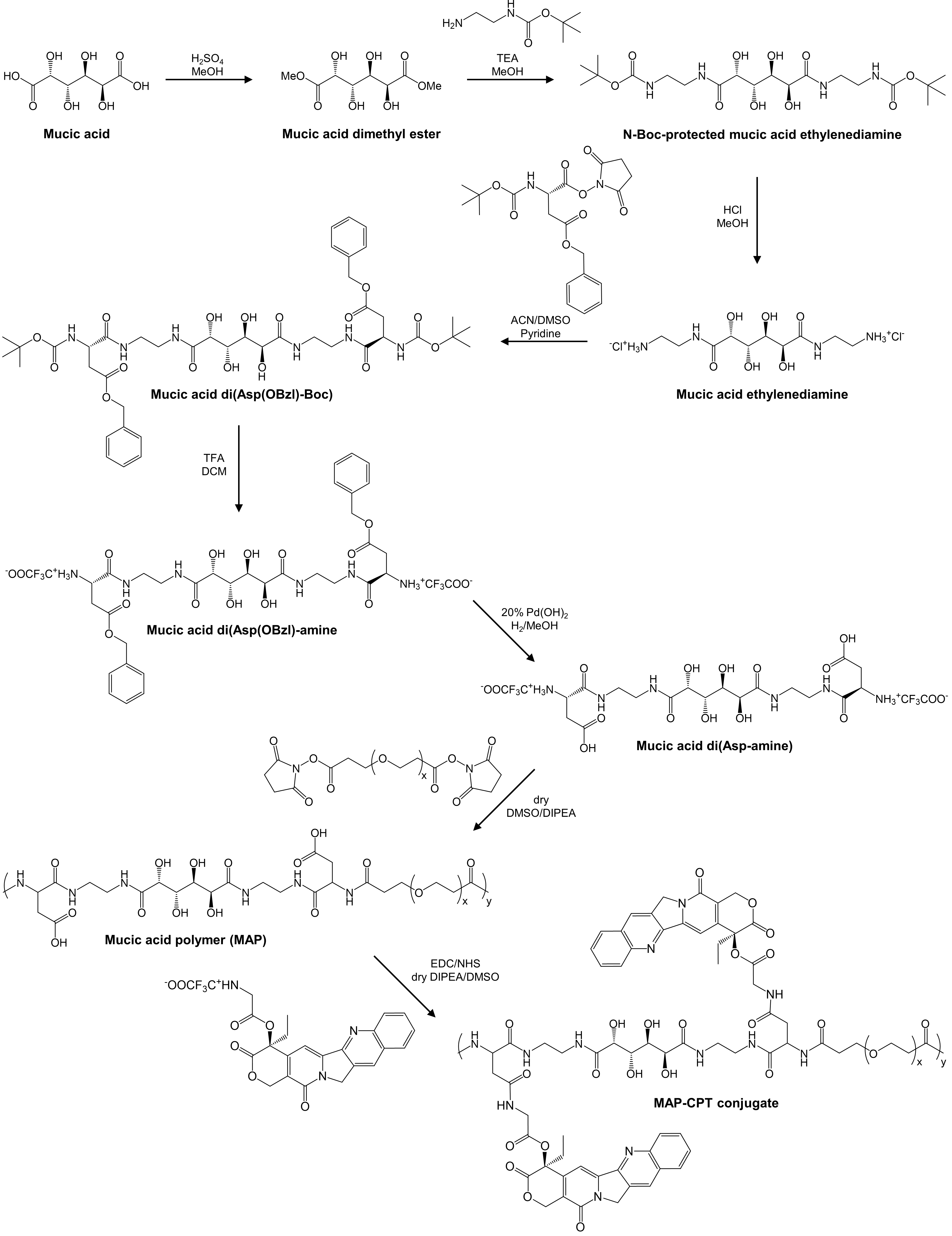
**

**Fig. S1.** Synthesis of MAP polymer followed by conjugation of CPT to prepare MAP-CPT conjugate. x ~ 82 for 3.4kDa PEG; y ~ 20 for material used in this study.


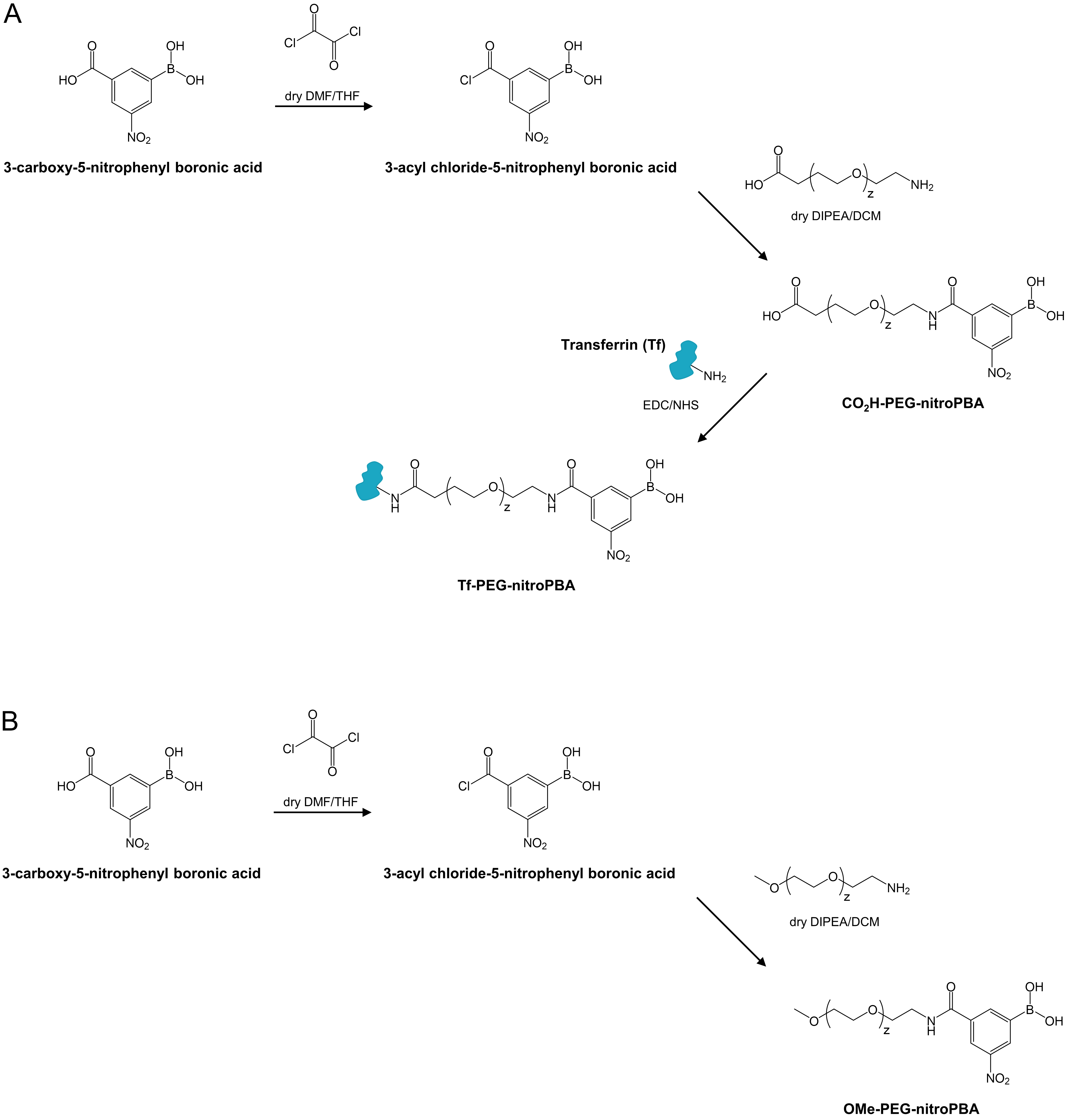


**Fig. S2.** Synthesis of nitroPBA conjugates. (**A**) Tf-PEG-nitroPBA. (**B**) OMe-PEG-nitroPBA. z ~ 120 for 5kDa PEG.


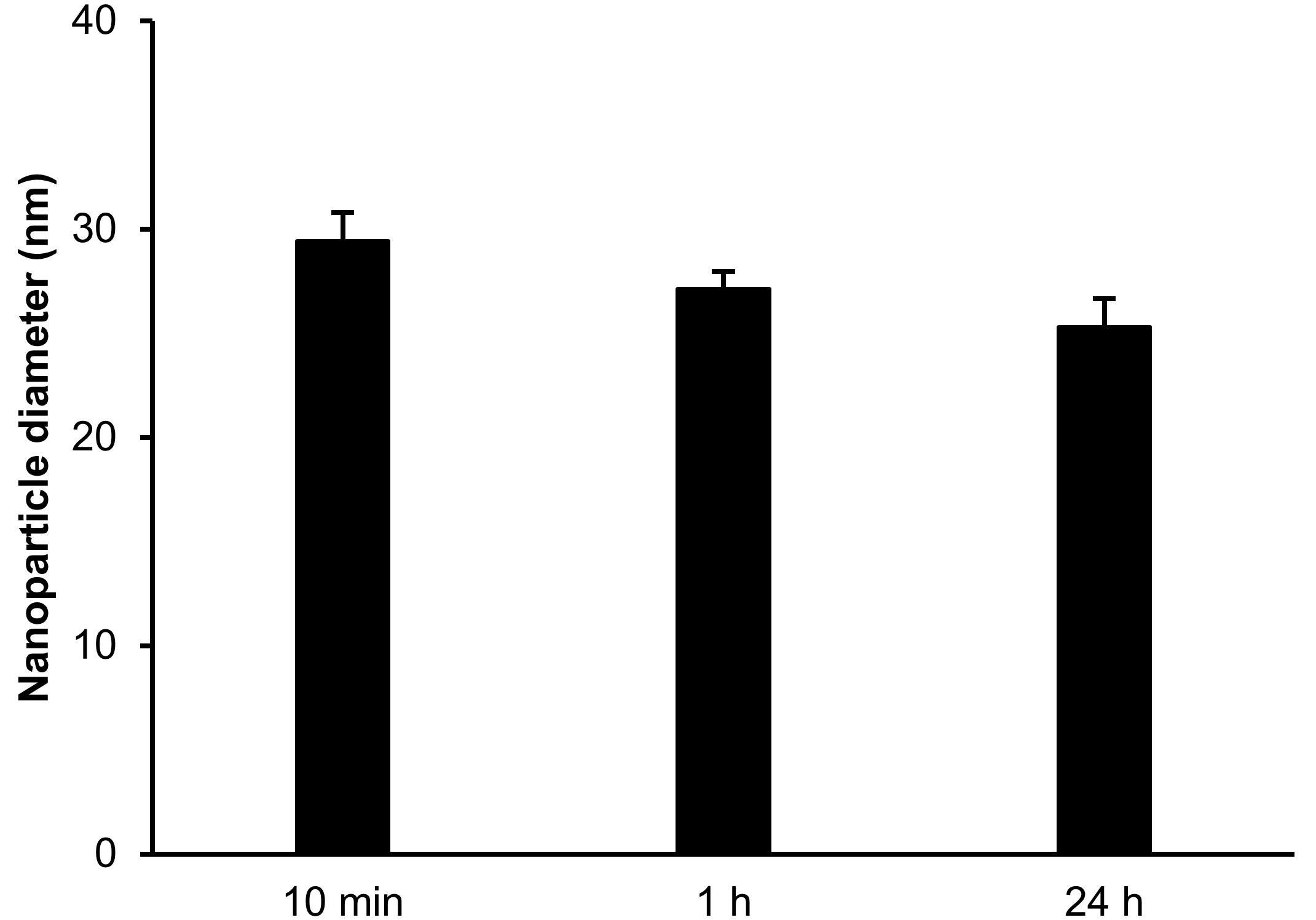


**Fig. S3.** TfR-targeted MAP-CPT nanoparticle diameter over time. No aggregation or increase in size was evident in the sample after 24 h, indicating the nanoparticles were not crosslinking due to introduction of multiple nitroPBA-PEG groups per Tf. Error bars indicate one standard deviation from the mean. Data shown are the average of 5 measurements ±1 SD.


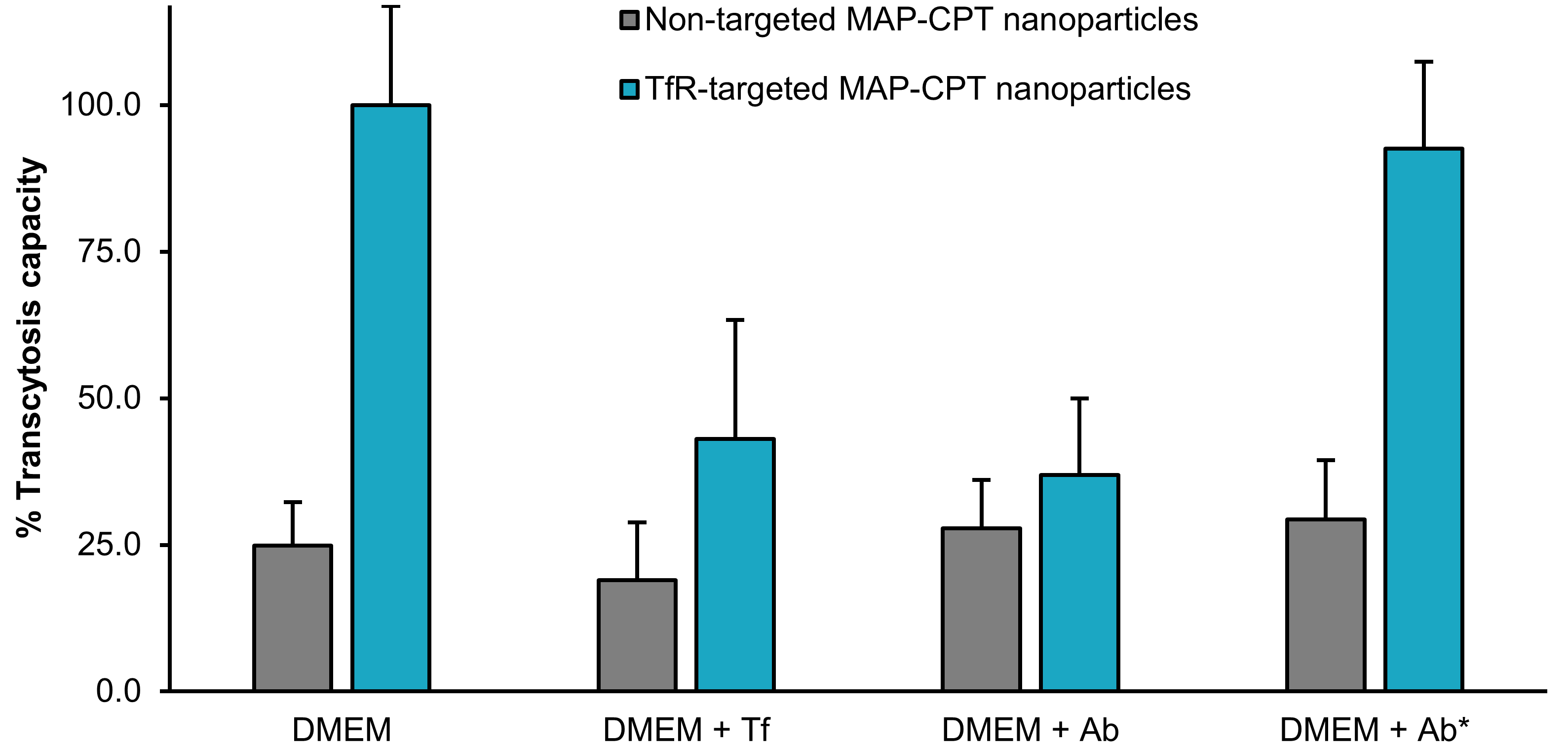


**Fig. S4.** Apical to basal transport of non-targeted and TfR-targeted MAP-CPT nanoparticles in model BBB. TfR-targeted (blue) and non-targeted (gray) nanoparticles were added to apical wells in either serum-free DMEM (DMEM), or in the presence of 2.5 mg/mL Tf (DMEM + Tf), equimolar high-affinity anti-TfR Ab (DMEM + Ab) or equimolar pH-dependent anti-TfR Ab (DMEM + Ab^*^). Data shown are the average of 4 wells for each group. Error bars indicate SE.

^*^Anti-TfR Ab with high affinity at pH 7.4 and reduced affinity at pH 5.5.

**
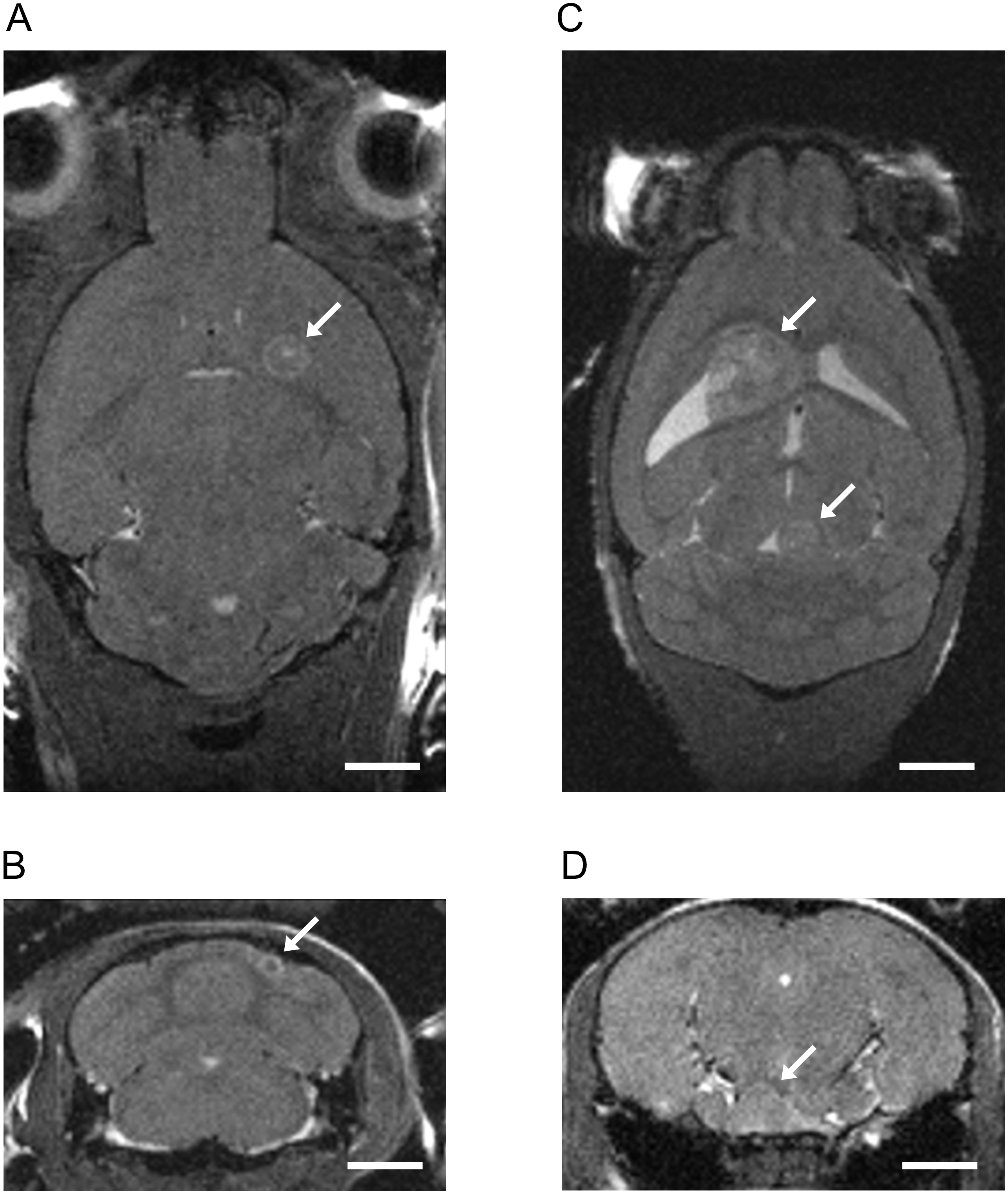
**

**Fig. S5.** Metastatic brain tumors imaged by MRI following IV injection of BT474-Gluc cells. Intracerebral (**A**,**B**,**C**) and leptomeningeal metastases were detected (**D**). Most intracerebral metastases were located in the cerebrum (**A**), with occasional metastases in the cerebellum (**B**). Multifocal metastases were occasionally observed (**C**). Leptomeningeal metastases most commonly grew in the subarachnoid space (**D**). Scale bar, 2 mm.

**
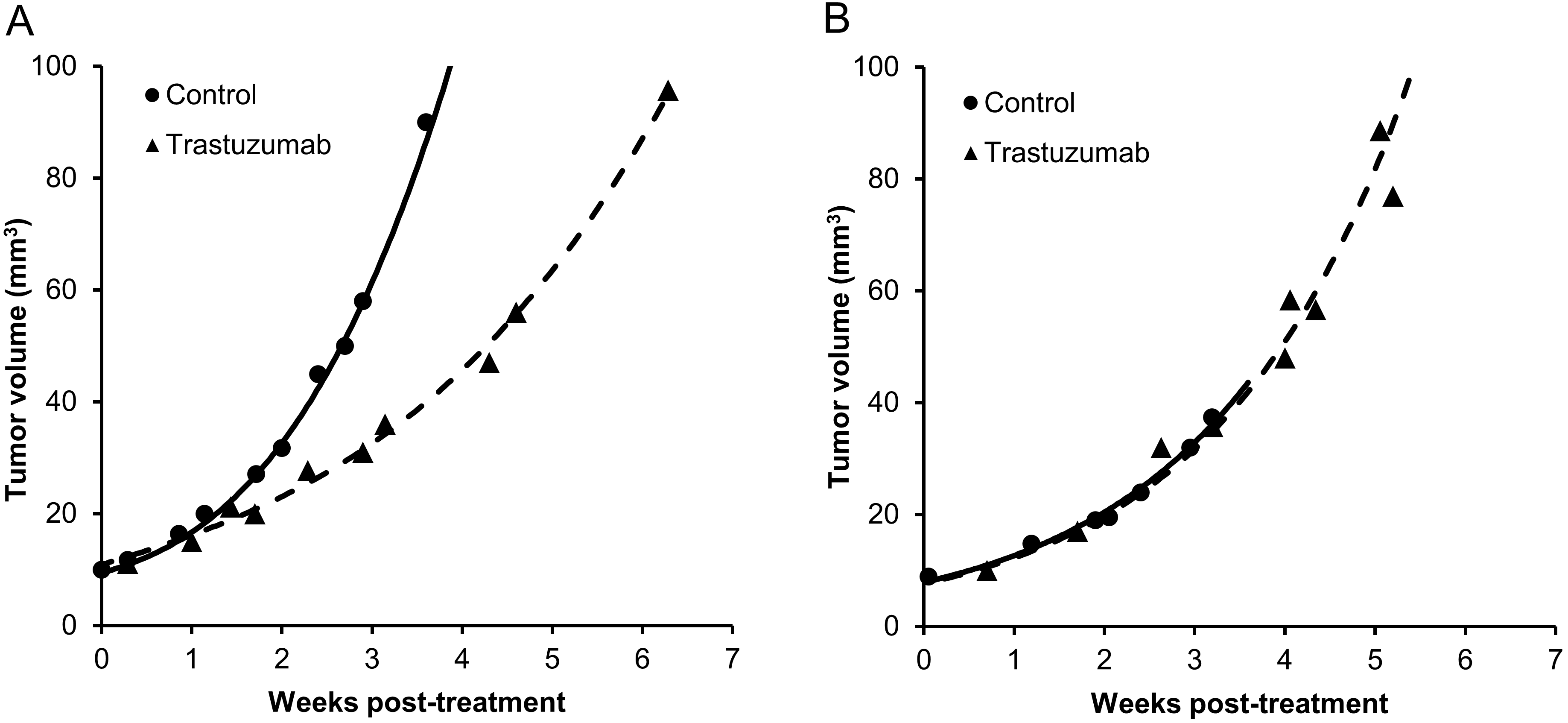
**

**Fig. S6.** Effect of anti-HER2 therapy on HER2-positive BT474-Gluc breast cancer brain metastases established in Rag2^-/-^;Il2rg^-/-^ mice. Trastuzumab at 5 mg/kg was administered twice weekly via intravenous tail vein injection, and treatment was initiated when tumors reached 10 mm^3^ in volume. MRI was used to monitor brain tumor size and response to treatment. Tumors established by IC injection (**A**) showed significant delay in tumor progression, whereas those established by IV injection (**B**) did not.


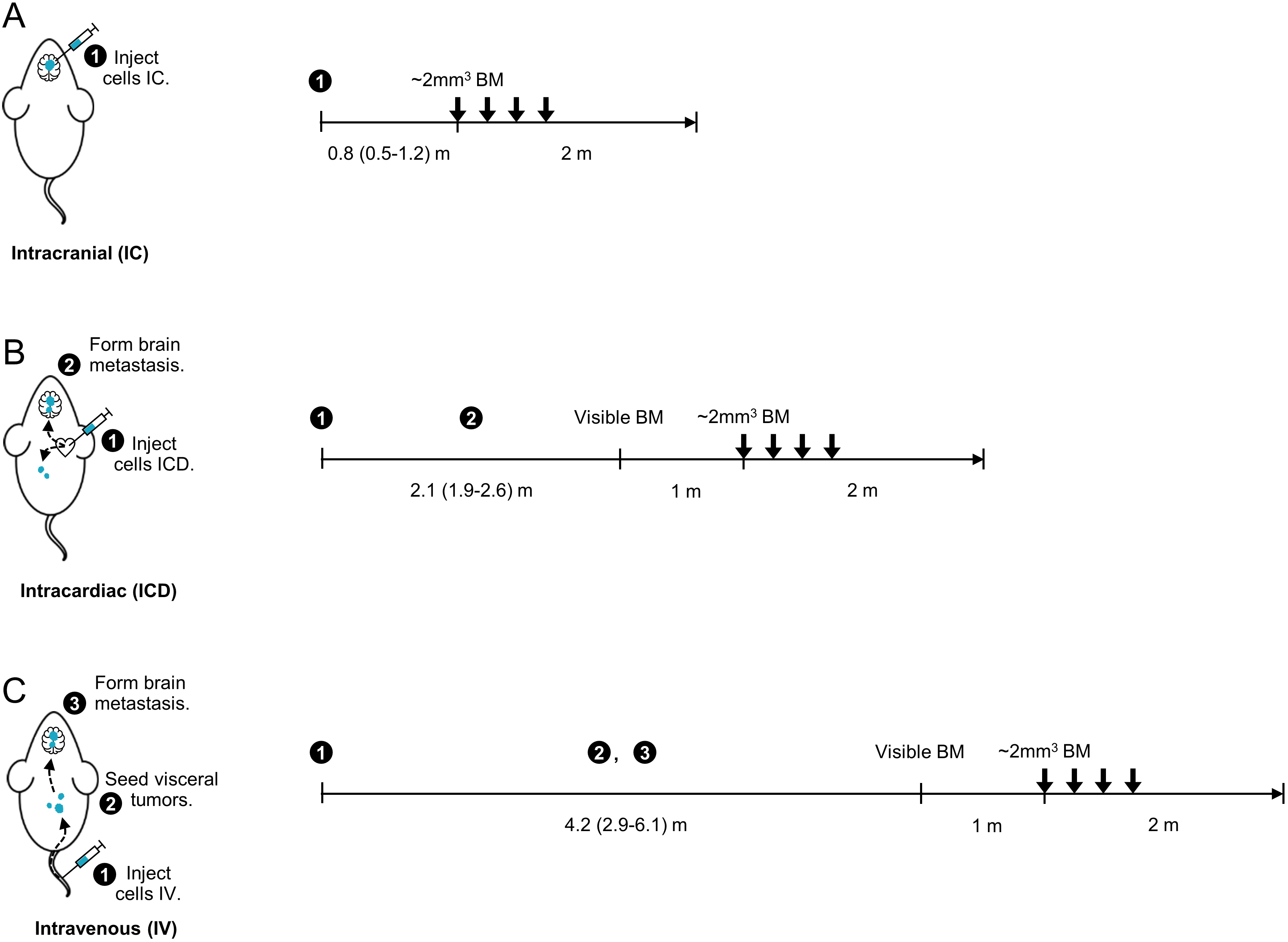


**Fig. S7.** Detailed illustration of intracranial (**A**), intracardiac (**B**), and intravenous (**C**) breast cancer brain metastasis models and timelines for efficacy study. Numbers below timeline indicate mean (range) time in months to establishment of visible brain metastases (BM; ~0.2 mm^3^ in volume) by MRI. Thick arrows denote treatment schedule for the study, with 4 weekly doses administered once tumors reached ~2 mm^3^ in volume.

**
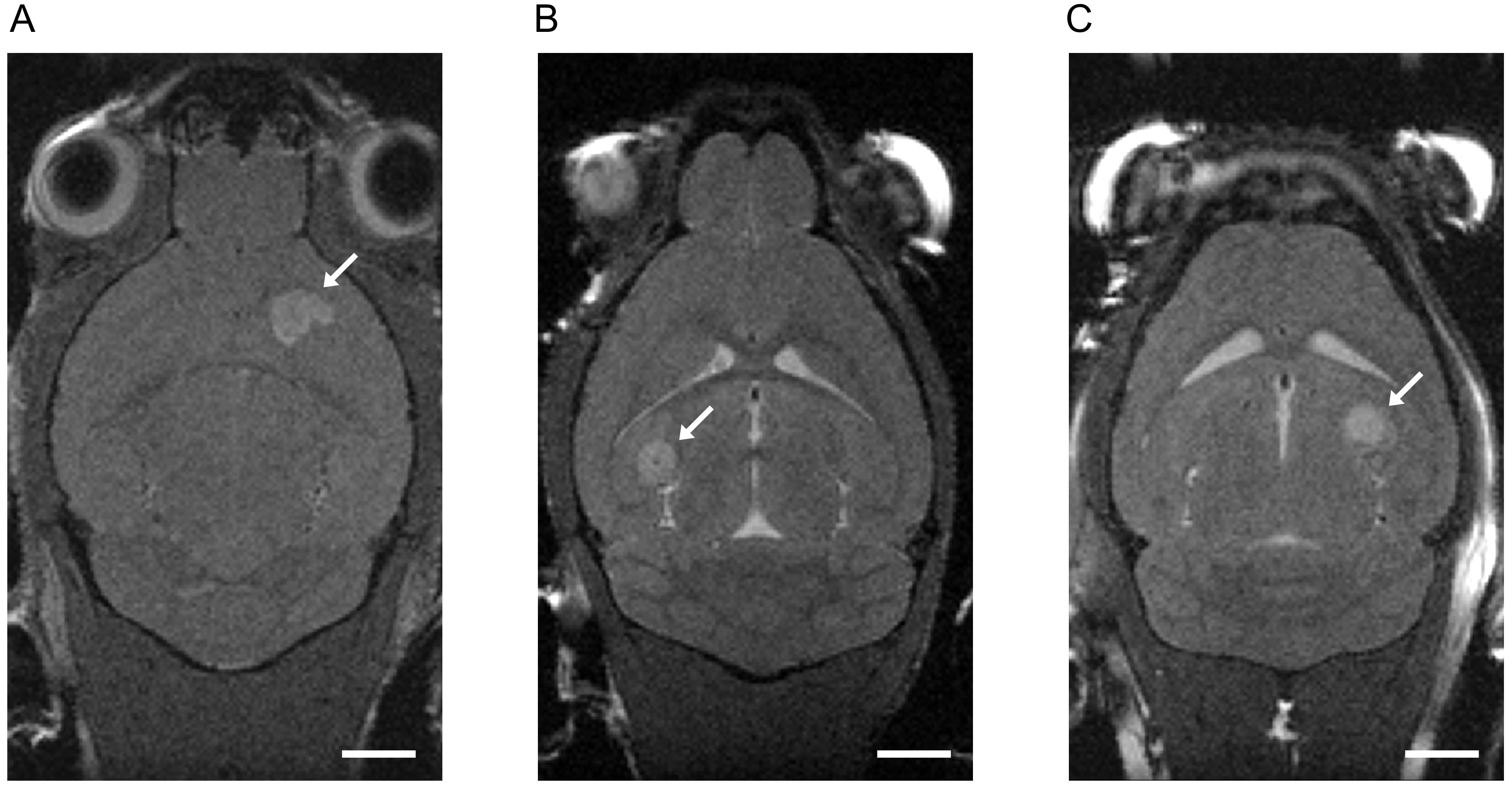
**

**Fig. S8.** Brain tumors imaged by MRI for intracranial (**A**), intracardiac (**B**), and intravenous (**C**) breast cancer brain metastasis models. Scale bar, 2 mm.


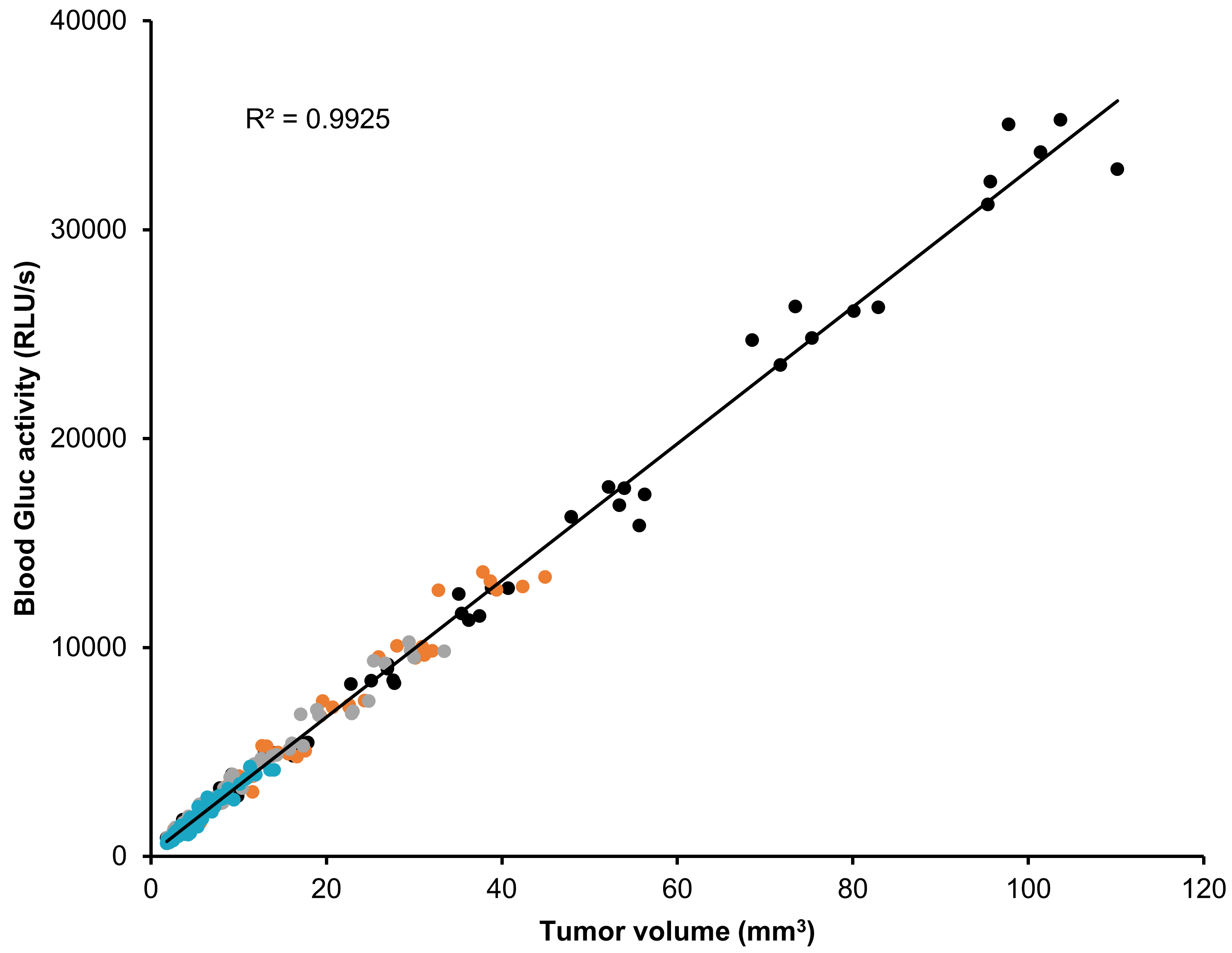


**Fig. S9.** Blood Gluc activity of IC-established tumors is correlated with tumor volume, as measured by MRI, for each treatment group. Blood Gluc activity is plotted against tumor volume for saline (black), CPT (orange), non-targeted MAP-CPT nanoparticle (gray), and TfR-targeted MAP-CPT nanoparticle (blue) treatment groups. Linear regression was performed using MATLAB.

**
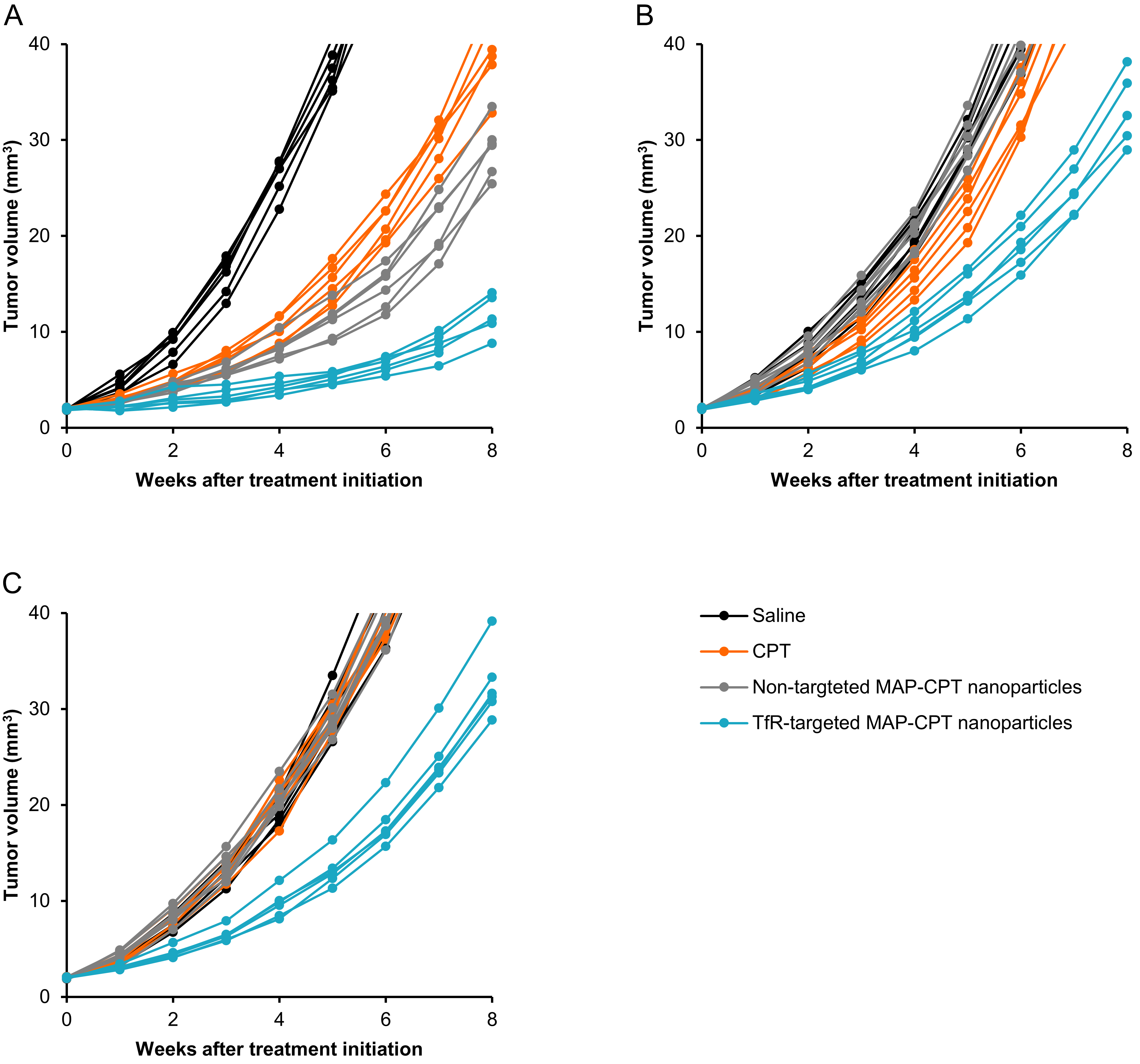
**

**Fig. S10.** Individual tumor growth curves of BT474-Gluc metastatic brain tumors treated with CPT (orange, 4 mg/kg), non-targeted MAP-CPT nanoparticles (gray, 4 mg CPT/kg), and TfR-targeted MAP-CPT nanoparticles (blue, 4 mg CPT/kg) compared to saline (black) when established by IC (**A**), ICD (**B**), and IV injection (**C**).


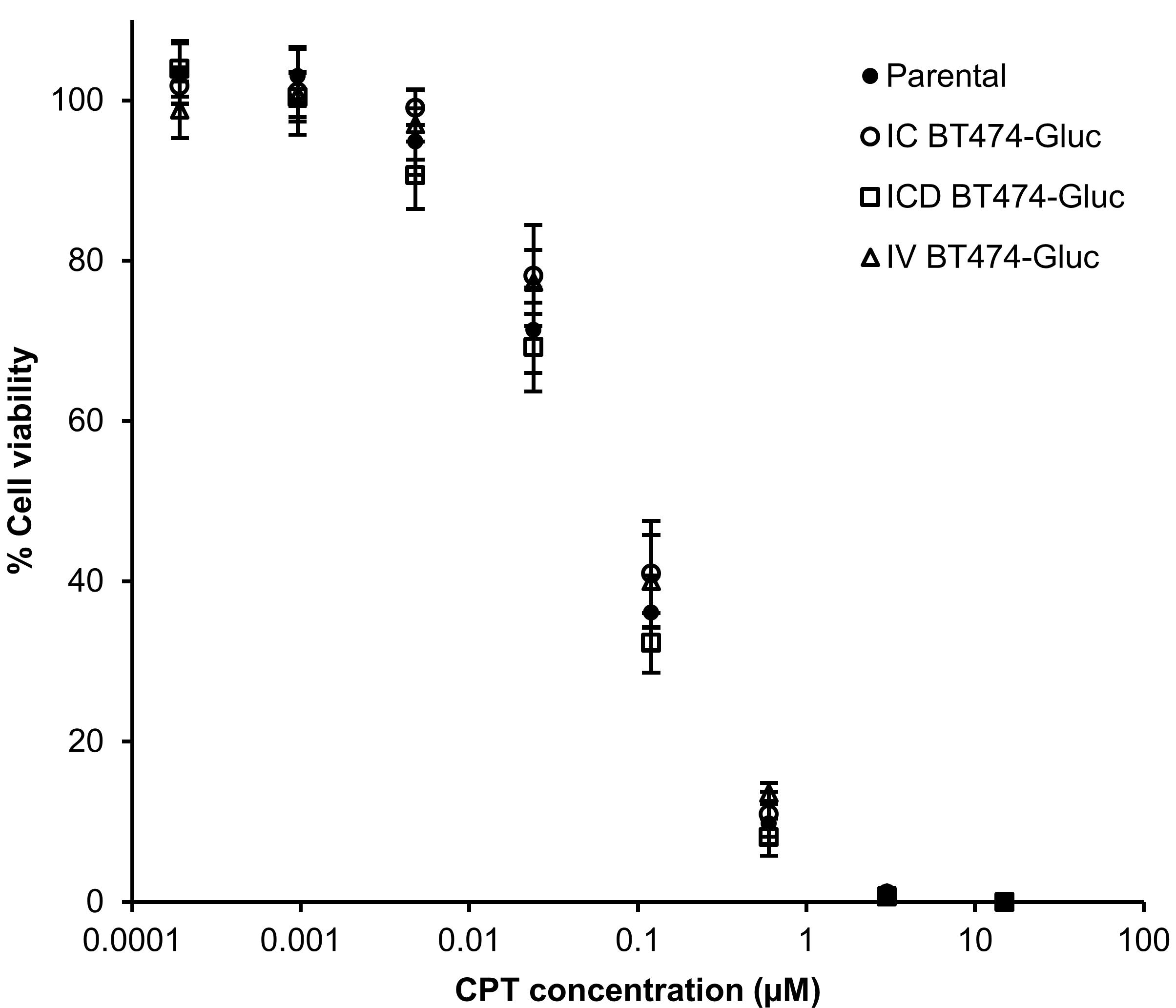


**Fig. S11.** BT474-Gluc cells isolated from brain tumors following IC- (circle), ICD- (square), and IV-establishment (triangle) as well as parental cells (solid circle) are similarly sensitive to CPT. Data shown are the average of 4 dose-response curves for each cell line. Error bars indicate SE.

**Supplemental Tables**

**Table S1.** Properties of MAP polymer and MAP-CPT polymer-drug conjugate.

| Material | Property |  |
| --- | --- | --- |
| MAP polymer | d*n*/dc (mL/g) | 0.14 |
|  | MW^*^ (kDa) | 68 |
|  | Polydispersity^†^ | 1.26 |
| MAP-CPT conjugate | Wt % CPT | 11.8 |

^*^MW, molecular weight determined as (*M*_w_ + *M*_n_)/2; *M*_w_, weight average molecular weight; *M*_n_, number average molecular weight.

^†^Polydispersity determined as *M*_w_ /*M*_n_.

**Table S2.** Nanoparticle formulations and characteristics.

| Formulation | Nanoparticle diameter, pH 7.4, nm | Zeta potential, pH 7.4, mV | Nanoparticle diameter, pH 5.5, nm | Zeta potential, pH 5.5, mV |
| --- | --- | --- | --- | --- |
| MAP-CPT nanoparticle | 37.8 ± 1.4 | -0.39 ± 0.78 | 38.2 ± 1.8 | -0.27 ± 0.84 |
| TfR-targeted MAP-CPT nanoparticle | 29.4 ± 1.2 | -1.32 ± 0.45 | 37.9 ± 1.3 | -0.51 ± 0.42 |
| Non-targeted MAP-CPT nanoparticle | 45.6 ± 1.7 | -0.57 ± 0.88 | 37.6 ± 1.9 | -0.43 ± 0.68 |

Data shown for hydrodynamic diameter and zeta potential are the average of 5 measurements ±1 SD.

**Table S3.** Metastatic ability of human BT474-Gluc breast cancer cells in Rag2^-/-^;Il2rg^-/-^ mice following IV injection.

| Brain | Lung | Bone | Liver | Ovary | Lymph | Other^*^ |
| --- | --- | --- | --- | --- | --- | --- |
| 22/24 | 24/24 | 6/8 | 16/17 | 24/24 | 24/24 | 19/24 |

Metastasis incidence provided by site per number of mice for which tissue type was analyzed. ^*^Other metastatic sites included kidney, salivary glands, and interscapular space.

**Table S4.** Anatomical locations of brain tumors for metastasis models.

| Metastasis model | Intracerebral (cerebrum) | Intracerebral (cerebellum) | Leptomeningeal |
| --- | --- | --- | --- |
| Intracranial (IC) | 24 | 0 | 0 |
| Intracardiac (ICD) | 18 | 4 | 2 |
| Intravenous (IV) | 19 | 3 | 2 |

**Table S5.** Antitumor efficacy in Rag2^-/-^;Il2rg^-/-^ mice bearing human BT474-Gluc breast cancer metastatic brain tumors established by IC injection.

|  | Mean tumor volume (mm^3^) | Median tumor volume (mm^3^) | *P* vs. saline |
| --- | --- | --- | --- |
| Saline | 101 | 100 | - |
| CPT (4 mg/kg) | 39 | 39 | 0.0022 |
| Non-targeted MAP-CPT nanoparticle (4 mg CPT/kg) | 29 | 30 | 0.0022 |
| TfR-targeted MAP-CPT nanoparticle (4 mg CPT/kg) | 12 | 12 | 0.0022 |

Data provided are mean and median tumor volumes at the end of the study. *P* values were calculated using the Wilcoxon-Mann-Whitney test.

**Table S6.** Antitumor efficacy in Rag2^-/-^;Il2rg^-/-^ mice bearing human BT474-Gluc breast cancer metastatic brain tumors established by ICD injection.

|  | Mean tumor volume (mm^3^) | Median tumor volume (mm^3^) | *P* vs. saline |
| --- | --- | --- | --- |
| Saline | 87 | 88 | - |
| CPT (4 mg/kg) | 69 | 71 | 0.0022 |
| Non-targeted MAP-CPT nanoparticle (4 mg of CPT/kg) | 87 | 89 | 0.9372 |
| TfR-targeted MAP-CPT nanoparticle (4 mg of CPT/kg) | 33 | 32 | 0.0022 |

Data provided are mean and median tumor volumes at the end of the study. *P* values were calculated using the Wilcoxon-Mann-Whitney test.

**Table S7.** Antitumor efficacy in Rag2^-/-^;Il2rg^-/-^ mice bearing human BT474-Gluc breast cancer metastatic brain tumors established by IV injection.

|  | Mean tumor volume (mm^3^) | Median tumor volume (mm^3^) | *P* vs. saline |
| --- | --- | --- | --- |
| Saline | 83 | 83 | - |
| CPT (4 mg/kg) | 86 | 86 | 0.5887 |
| Non-targeted MAP-CPT nanoparticle (4 mg of CPT/kg) | 84 | 84 | 0.9372 |
| TfR-targeted MAP-CPT nanoparticle (4 mg of CPT/kg) | 33 | 31 | 0.0022 |

Data provided are mean and median tumor volumes at the end of the study. *P* values were calculated using the Wilcoxon-Mann-Whitney test.

**Table S8.** *P* values for pairwise comparisons of uptake of therapeutics in brain metastases.

|  | IC-CPT^*^ | IC-Non^†^ | IC-TfR^‡^ | ICD-CPT^§^ | ICD-Non^¶^ | ICD-TfR^#^ | IV-CPT^\|\|^ | IV-Non^**^ | IV-TfR^††^ |
| --- | --- | --- | --- | --- | --- | --- | --- | --- | --- |
| IC-CPT | X | - | - | - | - | - | - | - | - |
| IC-Non | 0.8857 | X | - | - | - | - | - | - | - |
| IC-TfR | 0.0286 | 0.0286 | X | - | - | - | - | - | - |
| ICD-CPT | 0.0286 | 0.0286 | 0.0286 | X | - | - | - | - | - |
| ICD-Non | 0.0286 | 0.0286 | 0.0286 | 0.0571 | X | - | - | - | - |
| ICD-TfR | 0.8857 | 0.8857 | 0.0286 | 0.0286 | 0.0286 | X | - | - | - |
| IV-CPT | 0.0286 | 0.0286 | 0.0286 | 0.2286 | 0.0571 | 0.0286 | X | - | - |
| IV-Non | 0.0286 | 0.0286 | 0.0286 | 0.0571 | 0.9714 | 0.0286 | 0.2000 | X | - |
| IV-TfR | 0.8857 | 0.8571 | 0.0286 | 0.0286 | 0.0286 | 1.000 | 0.0286 | 0.0286 | X |

Values were calculated using the Wilcoxon-Mann-Whitney test.

^*^CPT, IC model; ^†^Non-targeted MAP-CPT nanoparticles, IC model; ^‡^TfR-targeted MAP-CPT nanoparticles, IC model; ^§^CPT, ICD model; ^¶^Non-targeted MAP-CPT nanoparticles, ICD model; ^#^TfR-targeted MAP-CPT nanoparticles, ICD model; ^||^CPT, IV model; ^**^Non-targeted MAP-CPT nanoparticles, IV model; ^††^TfR-targeted MAP-CPT nanoparticles, IV model.

**Table S9.** *P* values for pairwise comparisons of uptake of therapeutics in healthy brain tissue.

|  | IC-CPT^*^ | IC-Non^†^ | IC-TfR^‡^ | ICD-CPT^§^ | ICD-Non^¶^ | ICD-TfR^#^ | IV-CPT^\|\|^ | IV-Non^**^ | IV-TfR^††^ |
| --- | --- | --- | --- | --- | --- | --- | --- | --- | --- |
| IC-CPT | X | - | - | - | - | - | - | - | - |
| IC-Non | 0.5714 | X | - | - | - | - | - | - | - |
| IC-TfR | 0.0286 | 0.0286 | X | - | - | - | - | - | - |
| ICD-CPT | 0.0286 | 0.0286 | 0.0286 | X | - | - | - | - | - |
| ICD-Non | 0.0857 | 0.1714 | 0.0286 | 0.0286 | X | - | - | - | - |
| ICD-TfR | 0.0286 | 0.0286 | 0.4857 | 0.0286 | 0.0286 | X | - | - | - |
| IV-CPT | 0.8286 | 1.000 | 0.0286 | 0.0571 | 0.2857 | 0.0286 | X | - | - |
| IV-Non | 0.2000 | 0.4857 | 0.0286 | 0.0286 | 0.4857 | 0.0286 | 0.5714 | X | - |
| IV-TfR | 0.0286 | 0.0286 | 0.9714 | 0.0286 | 0.0286 | 0.8857 | 0.0286 | 0.0286 | X |

Values were calculated using the Wilcoxon-Mann-Whitney test.

^*^CPT, IC model; ^†^Non-targeted MAP-CPT nanoparticles, IC model; ^‡^TfR-targeted MAP-CPT nanoparticles, IC model; ^§^CPT, ICD model; ^¶^Non-targeted MAP-CPT nanoparticles, ICD model; ^#^TfR-targeted MAP-CPT nanoparticles, ICD model; ^||^CPT, IV model; ^**^Non-targeted MAP-CPT nanoparticles, IV model; ^††^TfR-targeted MAP-CPT nanoparticles, IV model.
